# Multi-omics Comparative Analysis Reveals Multiple Layers of Host Signaling Pathway Regulation by the Gut Microbiota

**DOI:** 10.1128/mSystems.00107-17

**Published:** 2017-10-24

**Authors:** Nathan P. Manes, Natalia Shulzhenko, Arthur G. Nuccio, Sara Azeem, Andrey Morgun, Aleksandra Nita-Lazar

**Affiliations:** a Laboratory of Immune System Biology, National Institute of Allergy and Infectious Diseases, National Institutes of Health, Bethesda, Maryland, USA; b College of Veterinary Medicine, Oregon State University, Corvallis, Oregon, USA; c College of Pharmacy, Oregon State University, Corvallis, Oregon, USA; MIT

**Keywords:** cell signaling, host-microbiome interaction, microbiota, multi-omics, proteomics, systems biology, transcriptomics

## Abstract

The bodies of mammals are hosts to vast microbial communities composed of trillions of bacteria from thousands of species, whose effects on health and development have begun to be appreciated only recently. In this investigation, an integrated analysis combining proteomics and transcriptomics was used to quantitatively compare the terminal ilia from conventional and germfree mice. Female and male mice responded similarly to the microbiota, but C57BL/10A mice responded more strongly than BALB/c mice at both the transcriptome and proteome levels. The microbiota primarily caused upregulation of immunological pathways and downregulation of metabolic pathways in the conventional mice. Many of the affected pathways were altered only at either the transcriptome or proteome level. Of the pathways that were affected at both levels, most were affected concordantly. The discordant pathways were not principally involved in the immune system but instead were related to metabolism, oxidative phosphorylation, protein translation, transport, and turnover. To broaden the discovery of affected host pathways, a meta-analysis was performed using intestinal transcriptomics data from previously published studies of germfree versus conventional mice with diverse microbiota populations. Similar transcript-level responses to the microbiota were found, and many additional affected host pathways were discovered.

**IMPORTANCE** Multiple host pathways were affected by its adaptation to the microbiota. We have found significant transcriptome-proteome discordance caused by the microbiota. This discovery leads to the definite conclusion that transcript-level analysis is not sufficient to predict protein levels and their influence on the function of many specific cellular pathways, so only analysis of combinations of the quantitative data determined at different levels will lead to a complete understanding of the complex relationships between the host and the microbiota. Therefore, our results demonstrate the importance of using an integrative approach to study host-microbiota interaction at the molecular level.

## Abstract (precis)

Multiple host pathways were affected by its adaptation to the microbiota. We have found significant transcriptome-proteome discordance caused by the microbiota. This discovery leads to the definite conclusion that transcript-level analysis is not sufficient to predict protein levels and their influence on the function of many specific cellular pathways, so only analysis of combinations of the quantitative data determined at different levels will lead to a complete understanding of the complex relationships between the host and the microbiota. Therefore, our results demonstrate the importance of using an integrative approach to study host-microbiota interaction at the molecular level.

## INTRODUCTION

The bodies of mammals are homes to vast populations of indigenous microbes. Each collection of microbes is called a microbiota, and each collective metagenome, sometimes referred to as a “second genome,” is called a microbiome. The human microbiome contains ~7,000 species and ~1,000,000 genes, and most of it is located in the gut (1). The microbiota is heterogeneous and dynamic: it changes with age, diet, and health status (2). Numerous aspects of host physiology are influenced by the gut microbiota, including the development of the immune system, drug metabolism, nutritional status, and pathological states such as obesity, diabetes, cardiovascular disease, chronic gastrointestinal disorders such as inflammatory bowel disease, and asthma (3, 4). The significance of resident microbe-microbe and host-resident microbe interactions as a factor for human health (5) and biomedical research (6) is becoming apparent. Consequently, interest in the human microbiota is rapidly growing, and the NIH Human Microbiome Project (HMP) and the Metagenomics of the Human Intestinal Tract (MetaHIT) programs were recently launched to coordinate efforts to elucidate its biology. In parallel, model systems are being used to study the influence of the microbiota on the host, and germfree (GF) mice are an important tool for studying the effects of the microbiota on the host’s physiology.

The characteristics of GF mice include dramatic physiological changes and metabolic and immune impairments (4). The cecum of GF mice is ~10-fold larger in volume than that of normal mice (7). In addition, GF mice have relatively small spleens and reduced Peyer’s patches (8). Our investigation focused on the terminal ileum (the final section of the small intestine) because it is a site of intense host-microbiota interaction frequently affected in humans during intestinal infections and autoimmune and inflammatory bowel disease (9–11). The terminal ileum also exhibits a response to the microbiota that is stronger than that seen with other locations of the intestine, and the terminal ileum displays concordant changes in mRNA and cytokine levels for the key T cell subsets (12). At the cellular level, the terminal ileum is highly heterogeneous; is composed of enterocytes, goblet cells, Paneth cells, enteroendocrine cells, microfold cells, stem cells, fibroblasts, blood and lymphatic vessels, lymphoid nodules, immune cells, smooth muscle, adipose tissue, and neurons; and contains very abundant Peyer’s patches (13). We have previously shown that the ilea of GF mice have greatly reduced immune cell populations (14).

System-level studies comparing GF, conventionally raised (C), and conventionalized animals have focused on gene expression analysis using DNA microarrays and next-generation sequencing. Only a small number of studies have used proteomics to compare GF, conventional, and conventionalized animals. The metaproteome of the microbiota has been investigated (15–19). Proteomics was used to compare the intestines of conventional, monoassociated, and GF pigs (20, 21), to study the ceca of GF and conventionalized chickens (22), to study the liver and proximal colon of GF and conventionalized mice (23), to compare the liver and kidneys of conventional, GF, and antibiotics-treated mice (24), and to examine the effect of both gut and eye microbiota on immunity to ocular infections (25). Additionally, characteristics of histone modification within the proximal colon, liver, and white adipose tissue were compared between conventional, conventionalized, and GF mice (26). However, proteomics has never before been used to compare the gastrointestinal tracts of conventional and GF mice.

Here we quantitatively compared the ilea of conventional and GF mice at both the transcriptomic and proteomic levels and performed a comprehensive, comparative pathway analysis. Numerous studies have identified host sex-mediated (27–29) and strain-mediated (30–32) differences in host-microbiota interactions, so we included female and male mice from two strains (BALB/c and C57BL/10A) in our study. We discovered pathway differences between the GF and conventional mice at both the transcript and protein levels. The effects of the microbiota on female and male mice were similar, but the magnitude of the responses was larger for the C57BL/10A mice than for the BALB/c mice. We were able to conclude that transcript-level analysis is not sufficient to predict protein levels and their influence on many specific cellular signaling pathways. We also performed a meta-analysis (M) using previously published transcriptomics data sets from comparisons of conventional and GF mouse whole-tissue intestine samples to broaden our analysis to more-diverse microbiota effects on hosts.

## RESULTS AND DISCUSSION.

### The ileal transcriptome and proteome adapt to the microbiota.

**FIG 1:**
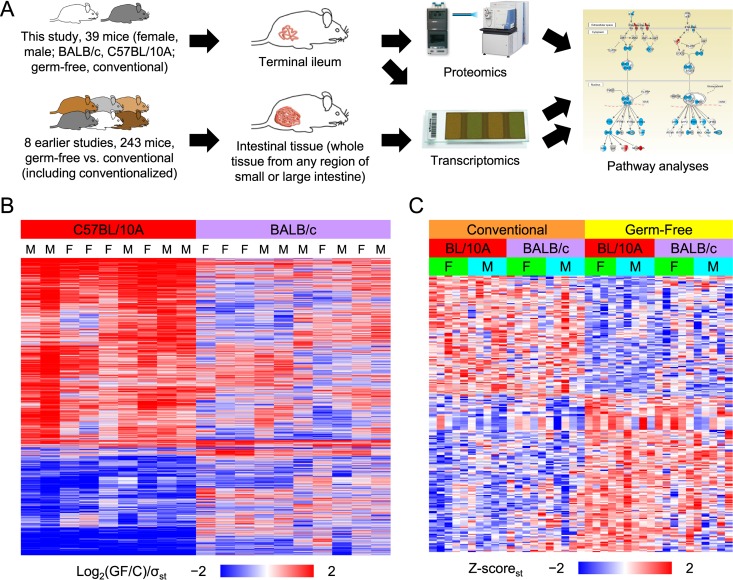
Study overview. (A) Terminal ilea from conventional and GF mice were quantitatively compared to produce the "ileum data set." Transcriptomics was used with DNA microarrays to produce the "transcriptome data subset," and the same ileum samples were also quantitatively compared using shotgun mass spectrometry to produce the “proteome data subset.” In addition, the “meta-analysis data set” was produced by analyzing microarray data from previous publications (the meta-analysis did not use any of the ileum data set). Genes significantly affected by germ status were analyzed using Ingenuity pathway analysis. GAS, gamma interferon (IFN-y)-activating sequence; ISRE, IFN-stimulated response element; TC-PTP, T-cell protein tyrosine phosphatase. (B) The transcriptome data subset was analyzed using an HCA (both rows and columns were clustered; gray = missing value). Each row depicts a microarray probe that was significantly affected by germ status (*n* = 3,817), and each column depicts an array. Each array was used to analyze two ilea (one conventional and 1 GF; sex and strain matched). Each standard deviation (σ_st_) was calculated across the columns independently for each strain using the log_2_-transformed GF/C ratios. F, female; M, male. (C) The proteome data subset was analyzed using an HCA (only rows were clustered; gray = missing value). Each row depicts a protein group that was significantly affected by germ status (*n* = 242), and each column depicts a mouse. Each *z* score was calculated across the columns independently for each strain using the log_2_-transformed abundance values.

To examine the effect of the microbiota on the mouse ileum, we performed a comparative analysis at the transcriptomic and proteomic levels (Fig. 1A). The abundances of many gene products were statistically significantly affected by germ status (3,063 and 219 genes in the transcriptome [T] and proteome [P] data subsets, respectively). We treated the germfree status as the perturbed state and therefore compared GF mice to conventional mice throughout the analysis, defining “upregulated” (up) as corresponding to GF/C ratio values of >1 and “downregulated” (down) as corresponding to GF/C ratio values of <1.

The ileum transcriptome data were analyzed to discover transcripts that were affected by germ status (see Tables S1 and S2 in the supplemental material). A hierarchical cluster analysis (HCA) partitioned the probe-level data into two clusters: upregulated probes (approximately the top two-thirds), and downregulated probes (approximately the bottom third) (Fig. 1B). Most of the probes were also affected by strain. Specifically, germ status had a stronger effect on the transcriptome of C57BL/10A mice than on that of BALB/c mice, and these effects were largely sex independent (Fig. 1B). The ileum transcriptome data were also analyzed using InfernoRDN to discover transcripts affected by sex and/or strain (see Fig. S1B in Text S1 in the supplemental material) (Table S3). The effect of mouse strain on the transcriptome was significant and largely independent of germ status (see Fig. S1A and B in Text S1). These results are consistent with earlier discoveries of strain-mediated differences in transcriptomes and proteomes of animal tissues. The hepatic transcriptome and proteome displayed significant variance across 97 mouse strains (33), and a similar result was found in a multi-omic study of liver from two rat strains (34). Additionally, numerous studies have identified host strain-mediated differences in host-microbiota interactions in the composition of the microbiota across 113 mouse strains (30); in Paneth cell population, antimicrobial peptide composition, and microbiota composition (31); or in microbiota composition and the colonic mucosa transcriptome (32).

10.1128/mSystems.00107-17.2 TABLE S1  Raw microarray data from the transcriptome data subset. The unadjusted reporter spot foreground and background intensity values (median pixel intensity) from the TXT files were produced by the use of Agilent Feature Extraction software. Download TABLE S1, XLSX file, 19.4 MB. Copyright © 2017 Manes et al. 2017 Manes et al. This content is distributed under the terms of the Creative Commons Attribution 4.0 International license.

10.1128/mSystems.00107-17.3 TABLE S2  BRB-ArrayTools results from the analysis of the transcriptome data subset. After the intra-array LOWESS normalizations, the data were anti-Log2 transformed to produce the GF/C values. Download TABLE S2, XLSX file, 13.6 MB. Copyright © 2017 Manes et al. 2017 Manes et al. This content is distributed under the terms of the Creative Commons Attribution 4.0 International license.

10.1128/mSystems.00107-17.1 TEXT S1  Figures S1 to S10. Download TEXT S1, DOCX file, 3.9 MB. Copyright © 2017 Manes et al. 2017 Manes et al. This content is distributed under the terms of the Creative Commons Attribution 4.0 International license.

10.1128/mSystems.00107-17.4 TABLE S3  InfernoRDN results from the analysis of the transcriptome data subset. The abundance values were log_2_ transformed and LOESS normalized. Download TABLE S3, XLSX file, 18.9 MB. Copyright © 2017 Manes et al. 2017 Manes et al. This content is distributed under the terms of the Creative Commons Attribution 4.0 International license.

The ileum proteome data were analyzed to discover protein groups that were affected by germ status, sex, and/or strain (Table S4). An HCA was used to analyze the protein groups significantly affected by germ status, and the rows partitioned into two clusters of roughly equal sizes (Fig. 1C): a downregulated (top half) protein group and an upregulated (bottom half) protein group. In addition to being affected by germ status, some of these protein groups were also affected by mouse strain. As with the transcriptome data (Fig. 1B), germ status had a stronger effect on the C57BL/10A proteome than on the BALB/c proteome. Interestingly, the effect was milder in the proteome than in the transcriptome.

10.1128/mSystems.00107-17.5 TABLE S4  InfernoRDN results from the analysis of the proteome data subset. The protein group abundance values were normalized and not log_2_ transformed. The Invariant (P; up + down) rows are highlighted in orange. Download TABLE S4, XLSX file, 2.2 MB. Copyright © 2017 Manes et al. 2017 Manes et al. This content is distributed under the terms of the Creative Commons Attribution 4.0 International license.

HCAs were also performed using the set of protein groups that were significantly affected by the other experimental parameters (see Fig. S1C and D in Text S1). As with the transcriptome data (see Fig. S1A and B in Text S1), mouse strain had a strong effect on the ileum proteome. We discovered that 63 proteins were both significantly affected by germ status and significantly unaffected by both sex and strain. An HCA of these proteins was performed (see Fig. S1D in Text S1), and the rows partitioned into two clusters of roughly equal sizes: an upregulated protein group and a downregulated protein group. We observed that the highly expressed genes were more frequently identified and quantified at the protein level and were more frequently classified as significantly affected by germ status at both the transcript and protein levels (see Fig. S2 in Text S1). Our proteome data subset was compared to a quantitative shotgun proteomics data set from a previous report (10 male GF C57BL/6 mice; at 57 to 62 days of age, 5 were conventionalized and the other 5 were mock conventionalized; proximal colons were harvested 14 days later) (23). The genes that were significantly affected by germ status within both sets of data were used for a correlation analysis, and the correlations were statistically significant (see Fig. S3A in Text S1).

**FIG 2:**
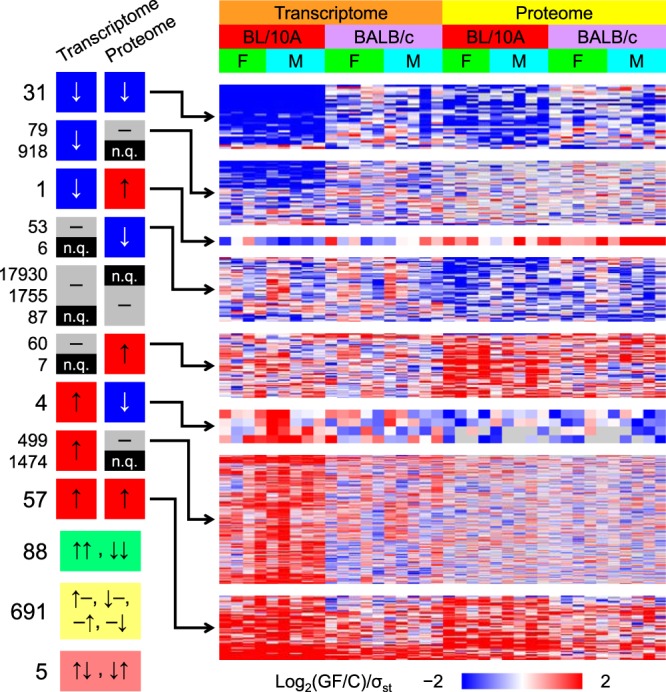
Concordance across the transcriptome and proteome data subsets. Genes were either upregulated due to germ status (red, GF/C > 1, *q*_min_ < 0.1), downregulated (blue, GF/C < 1, *q*_min_ < 0.1), unaffected (gray, *q*_min_ ≥0.1), or unquantified (n.q.; black). Tallies were used to quantify the overall levels of concordancy (light green) and discordancy (light yellow and red). The overlapping, significantly affected genes were analyzed using HCAs (only rows were clustered; gray = missing value). Each row depicts a gene, and each column depicts a pair of mice (1 GF, one conventional, sex and strain matched, from one microarray; same pairing for the proteome and transcriptome columns). Each standard deviation (σ_st_) was calculated across the columns independently for each data subset and mouse strain using the log_2_-transformed GF/C ratios.

The significantly affected genes in the transcriptome and proteome data subsets were tallied and analyzed using HCAs (Fig. 2). Most of the genes that were quantified within both data subsets and significantly affected within at least one of them were unaffected within the other. However, among the genes that were significantly affected within both data subsets, the effects were much more likely to be concordant than discordant. Data from a correlation analysis showed a highly significant correlation (see Fig. S3B in Text S1). This observation is consistent with numerous reports of moderate correlation of transcriptome and proteome abundance ratios from analyses of perturbed versus control samples (35). Germ status had a relatively strong effect on the C57BL/10A mice compared to the BALB/c mice, similarly to the results depicted in Fig. 1B and C.

### Adaptation of the ileum to the microbiota primarily affects metabolism and the immune system.

The overlapping genes from the transcriptome and proteome data that were significantly affected by germ status were used to produce interset concordant and discordant gene sets (described in detail in Materials and Methods; a total of 30 gene sets were produced). Ingenuity pathway analysis (IPA) was used to analyze these sets of genes to discover enriched biological pathways and other gene annotations (a total of 428 biological pathways were significantly enriched [gene annotation enrichment false-discovery rate {*q*_*e*_} = <0.1] in at least 1 of the 30 analyses; Tables 1, S5, S6, and S7). The pathway annotations overlapped more than the functional annotations (see Fig. S4 in Text S1). The microbiota caused upregulation of many immune system pathways in the conventional mice, and most of this was transcriptome-proteome concordant. It is likely that the microbiota causes an upregulation of intestinal immune system pathway gene expression in nonimmune cells. Also, we have previously reported that the microbiota causes an increase in immune cell populations in the terminal ileum (14), and this was likely a major cause of the observed upregulation. The microbiota also caused downregulation of many metabolic pathways in the conventional mice. Some of this dysregulation effect was transcriptome-proteome concordant, and some was discordant. The microbiota is known to perform numerous metabolic functions, and its absence likely causes the host to upregulate some of the corresponding pathways.

**TABLE 1:** Tally of the biological pathways that were significantly affected by germ status^a^

Gene set	Gene set description	No. of upregulated pathways (GF/C > 1)	No. of downregulated pathways (GF/C < 1)	No. of pathways showing intraset discordance
T	T (this study)	16	63	17
P	P (this study)	30	38	2
M	T (previous reports)	89	59	236
Concordant[T,P,M]	Concordant in T, P, and M	28	8	0
Concordant[T,P]	Concordant in T and P	39	32	1
Concordant[T,M]	Concordant in T and M	21	90	19
Discordant[T≠P]	In T and/or P; discordant between them	4	9	0
Discordant[T\P]	In T; discordant compared to P	4	0	0
Discordant[P\T]	In P; discordant compared to T	21	16	0
Invariant[P]	In P; unaffected by sex and strain	21	4	0

a T, transcriptome; P, proteome; M, meta-analysis.

10.1128/mSystems.00107-17.6 TABLE S5  Gene-level quantification data. Note that the transcriptomics data are from the BRB-ArrayTools analysis. Download TABLE S5, XLSX file, 2.1 MB. Copyright © 2017 Manes et al. 2017 Manes et al. This content is distributed under the terms of the Creative Commons Attribution 4.0 International license.

10.1128/mSystems.00107-17.7 TABLE S6  IPA canonical pathways. All 30 IPAs used the same IPA version (June 2016 release; Application Build 389077m; Content version 27821452). Download TABLE S6, XLSX file, 0.5 MB. Copyright © 2017 Manes et al. 2017 Manes et al. This content is distributed under the terms of the Creative Commons Attribution 4.0 International license.

10.1128/mSystems.00107-17.8 TABLE S7  IPA canonical pathway cross tab. These Ingenuity pathways were all significantly enriched (*q*_*e*_ < 0.1) in at least one of the 30 IPAs. For each of the 10 gene sets (columns), each pathway was classified as upregulated, downregulated, intraset discordant, or not significantly enriched. Download TABLE S7, XLSX file, 0.03 MB. Copyright © 2017 Manes et al. 2017 Manes et al. This content is distributed under the terms of the Creative Commons Attribution 4.0 International license.

**FIG 3:**
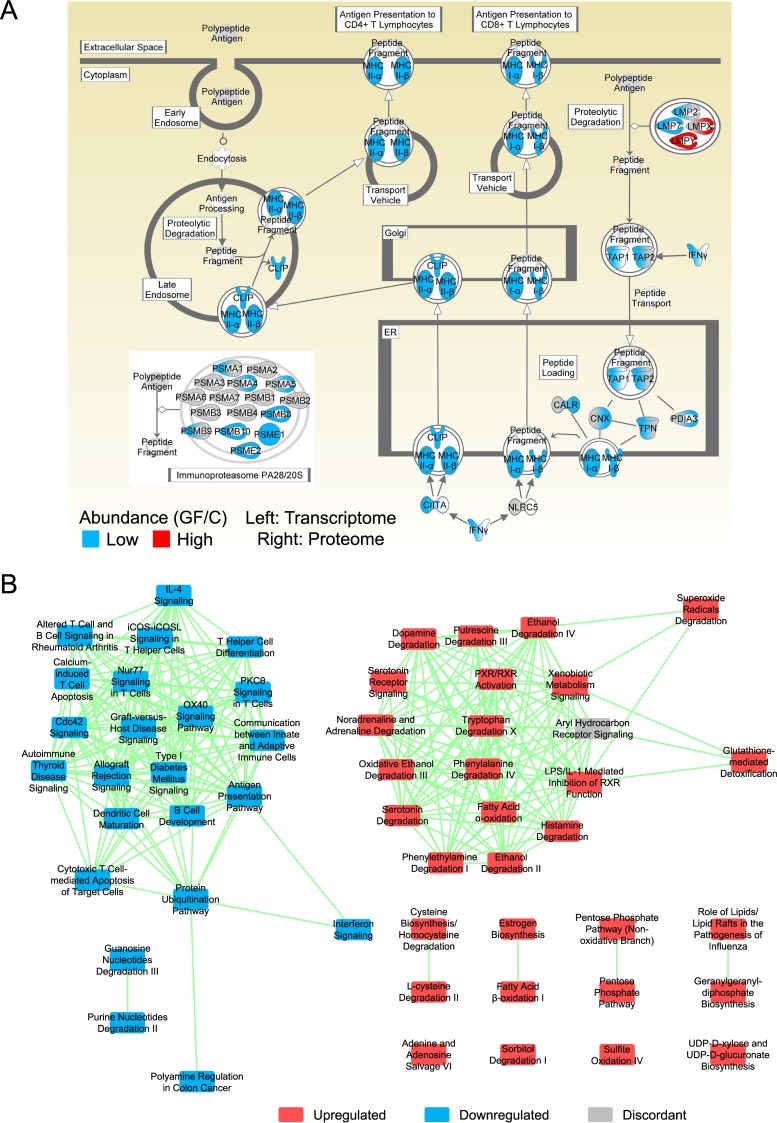
(A) The antigen presentation pathway was downregulated in the GF mice. Each pathway node was dually colored using the transcriptome (left) and proteome (right) data subsets. The immunoproteasome complex is from the protein ubiquitination pathway. The statistical significance data were *q*_e_ (transcriptome, downregulated) = 1.0E−14, *q*_e_ (proteome, downregulated) = 6.6E−10, *q*_e _(meta-analysis, downregulated) = 1.9E−3, *P*_c_ (transcriptome) = 3.4E−3, and *P*_c _(meta-analysis) = 3.9E−2. The proteome node-level intraset concordancy data were not statistically significant, but they were close (*P*_c_ = 6.5E−2; significance level = 0.05). CALR, calreticulin; CLIP, class II-associated invariant chain peptide; CNX, calnexin; ER, endoplasmic reticulum; MHC, major histocompatibility complex; TPN, tapasin. (B) The concordant[T,P] network of biological pathways formed two independent clusters related to the immune system (left) and metabolism (right). Each node depicts a significantly enriched biological pathway, and each edge depicts at least one instance of overlapping genes that were significantly affected by germ status. Pathways were classified as upregulated if the annotation enrichment was maximally significant by analyzing only the upregulated genes and likewise for the downregulated pathways. IL-4, interleukin-4.

Seventy-nine pathways were downregulated in the transcriptome and/or proteome data subsets, and many were immune system pathways. Twenty-two pathways were downregulated in both subsets, and almost all were immune system pathways such as the antigen presentation pathway (Fig. 3A). Forty-three pathways were upregulated in the transcriptome and/or proteome, and almost all were metabolism pathways. Only three pathways were upregulated in both: the glutathione-mediated detoxification pathway, the serotonin degradation pathway, and the xenobiotic metabolism signaling pathway. Thirty-two pathways were downregulated in the concordant[T,P] gene set, and most were immune system pathways. Thirty-nine pathways were upregulated in the concordant[T,P] gene set, and almost all were metabolism pathways.

Network analyses of the significantly enriched biological pathways were performed. Each used 1 of the 30 sets of genes significantly affected by germ status. For each gene set, five networks were examined (the minimum number of overlapping significantly affected genes per edge examined ranged from one to five). Network analysis of the proteome data subset (see Fig. S5A in Text S1) and of the concordant[T,P] gene set (Fig. 3B) resulted in two clusters. One was principally composed of downregulated immune system pathways, and the other was principally composed of upregulated metabolic pathways. Network analysis of the invariant[P, up] gene set produced a cluster of upregulated metabolic pathways (see Fig. S5B in Text S1) similar to the proteome and concordant[T,P] network results. This network might constitute the core set of pathways that are normally resistant to perturbation but are nevertheless altered by the microbiota.

### Ileal pathway adaptation to the microbiota is partially transcriptome-proteome discordant.

Transcriptome-proteome concordance is consistent with regulation occurring mainly at the transcriptional level. In contrast, gene products being significantly affected at the transcriptome level and not at the proteome level (discordant[T\P]), or the reverse (discordant[P\T]), would mean that regulation occurs principally via posttranscriptional or cotranslational regulation, targeted protein degradation, or protein secretion. There were 5 instances of opposite regulation at the transcriptomic and proteomic levels, 113 instances of significantly affected proteins and unaffected transcripts, and 578 instances of the reverse (Fig. 2). These data were analyzed to discover differentially regulated biological pathways.

**FIG 4:**
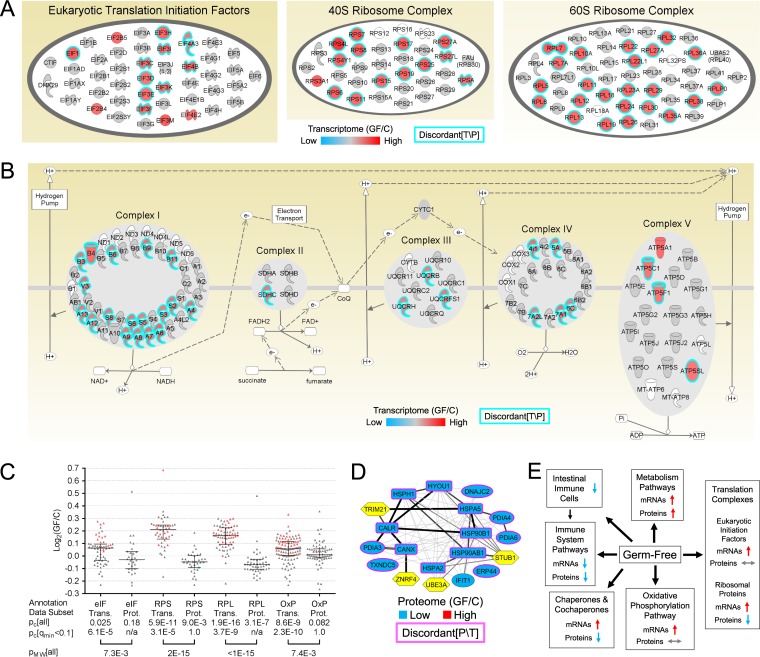
Intraset concordancy and transcriptome-proteome discordancy of gene products related to protein translation, OxP, and chaperone proteins. (A) The eIF and RP complexes displayed significant intraset concordancy (*P*_c_ [transcriptome] = 8.9E−16) and transcriptome-proteome discordancy (cyan outlines). (B) The OxP pathway also displayed significant intraset concordancy [*q*_e_ (transcriptome, up) = 3.4E−6, *P*_c_[transcriptome] = 9.3E−10] and transcriptome-proteome discordancy (cyan outlines). (C) All of the following categories of transcriptome and proteome data related to the four annotations were analyzed: eIFs, the 40S ribosomal complex (RPS), the 60S ribosomal complex (RPL), and the OxP pathway. Each triangle depicts a microarray probe (transcriptome [Trans.] data subset) or a protein group (quantified nonredundantly; proteome [Prot.] data subset). Red and blue triangles depict significantly increased and decreased gene products, respectively (gray triangles = not significantly affected). The lines indicate each median and interquartile range. Each binomial test either used all of the data or was restricted to just the significantly affected data, as indicated. n/a, not applicable. (D) A protein-protein interaction network was prepared using all of the chaperones (rectangles) and cochaperones (ovals) that were significantly affected by germ status within the proteome data subset, and the intraset concordance was significant (*P*_c_ = 6.1E−5). Also included are the E3 ubiquitin ligases (yellow hexagons) that have been reported to associate with chaperones and typically target misfolded/unfolded proteins. Edge weight data depict the protein-protein interaction confidence (increasing weight values depict low, medium, high, and very high confidence). (E) Summary of the effects on the GF mouse intestine. The decrease in immune cell populations was reported previously by Morgun et al. (14).

Four pathways were significantly enriched in the discordant[T\P] gene set, and all four were upregulated. Two of these pathways, the eukaryotic initiation factor 2 (eIF2) signaling and mechanistic target of sirolimus (mTOR) signaling (see Fig. S6 in Text S1) pathways, highly overlapped. These pathways included much of the protein translation machinery. The significantly affected genes annotated with the Ingenuity biofunctions “Processing of RNA” and “Translation” were analyzed, and a similar pattern was discovered (intratranscriptome concordancy and transcriptome-proteome discordancy; see Fig. S7 in Text S1). All of the significantly affected eIF and ribosomal protein (RP) genes were found to be upregulated in the transcriptome data subset, and almost all were transcriptome-proteome discordant (Fig. 4A). Intraset concordancy of all of the eIF, 40S RP, and 60S RP transcript- and protein-level data was analyzed, and it was discovered that the eIF, 40S RP, and 60S RP transcripts were upregulated, the eIF proteins were unaffected, and the 40S and 60S RPs were downregulated (Fig. 4C). Generally, such differences may result from changes in posttranscriptional regulation, mRNA export and localization, cotranslational regulation, protein secretion, or targeted protein degradation (35).

All RP genes and many eIF genes are known to be posttranscriptionally regulated via their 5′-terminal oligopyrimidine (5′TOP) tract (36, 37). In the inactive state, LARP1, TIA1, or TIAL1 is bound to the 5′TOP tract, which represses translation (38–40). LARP1 binding increases transcript stability, and TIA1 and TIAL1 binding targets the transcript to stress granules for mRNA triage. Mechanistic target of sirolimus (mTOR) complex 1 activation antagonizes repression of translation by LARP1, TIA1, and TIAL1. Posttranscriptional regulation by LARP1, TIA1, and/or TIAL1 may have caused the transcriptome-proteome discordancy that we observed. In addition, it is also possible that the microbiota caused a decrease in transcript stability via LARP1, TIA1, and/or TIAL1, resulting in the observed concordant downregulation of these transcripts in the conventional mice. A second 5′ untranslated region tract called the pyrimidine-rich translational element is also involved in mTOR-mediated translation upregulation of many RP transcripts (41), and it is possible that regulation at this tract was involved.

The other two discordant[T\P] pathways, the OxP and mitochondrial dysfunction pathways, also highly overlapped. The OxP pathway nodes were intratranscriptome concordant (all 31 were upregulated in the GF ilea), and they were all transcriptome-proteome discordant except for Atp5a1 (Fig. 4B). Mouse gene expression of OxP genes was found to be coregulated using a coexpression network analysis (data not shown), as previously reported (42). Intraset concordancy of all of the OxP transcript- and protein-level data was analyzed, and it was discovered that the OxP transcripts were upregulated and that the OxP proteins were unaffected (Fig. 4C).

Gene expression of OxP genes across 61 mouse tissues was previously reported to be coregulated (42). As with the eIF and RP genes, it is possible that the microbiota affects the transcription rate and/or transcript stability of OxP genes, resulting in the observed concordant downregulation of these transcripts in the conventional mice. Interestingly, we previously reported that transcripts encoding ribosomal and OxP proteins were significantly downregulated in the mouse ilea in response to treatment with either antibiotics or antibiotic-resistant microbes and that these effects were mediated by virulence factors and quorum sensing by antibiotic-resistant bacteria (14).

Thirty-seven pathways were significantly enriched in the discordant[P\T] gene set. Twenty-one were upregulated, 16 were downregulated, and none were intraset discordant. A network analysis of the 21 upregulated pathways resulted in a cluster of upregulated metabolic pathways (see Fig. S8A in Text S1). Thus, the proteomic analysis discovered many upregulated metabolism pathway elements that were missed by the transcriptomic analysis. Interestingly, of the 21 upregulated discordant[P\T] pathways, 12 were also upregulated invariant[P] pathways.

Fifteen of the 16 downregulated discordant[P\T] pathways contained chaperone proteins that were significantly affected by germ status. Therefore, the interset concordancy and intraset concordancy/discordancy of the chaperones and cochaperones in the transcriptome and proteome data subsets were analyzed. There were 310 (co)chaperone genes corresponding to 428 microarray probes in the transcriptome; 206 probes were upregulated, 222 probes were downregulated, and the results were found not to be significantly intraset concordant (*P*_c_ = 0.47). Forty-three (co)chaperone genes (44 microarray probes) were significantly affected by germ status in the transcriptome data subset. Of the 44 significantly affected probes, 31 were upregulated and 13 were downregulated, which represented significant intraset concordance (*P*_c_ = 9.6E−3). All 43 of the (co)chaperone genes affected at the transcript level were unaffected at the protein level, with one exception: Ifit1, an interferon-induced cochaperone, was downregulated at both levels.

In the proteome data subset, there were 107 (co)chaperone genes corresponding to 100 nonredundant quantified protein groups. Of the 100 nonredundant quantified protein groups, 37 were upregulated and 63 were downregulated, representing significant concordance (1.2E−2). Of the 100 nonredundant quantified protein groups, 15 were significantly affected by germ status, and all 15 were downregulated, representing significant intraset concordance (*P*_c_ = 6.1E−5). Many of these 15 proteins have been reported to directly associate with each other, and all 15 genes were unaffected in the transcriptome data subset except for the Ifit1 gene (Fig. 4D). Thus, the (co)chaperone transcripts and proteins were affected oppositely (transcripts upregulated and proteins downregulated). Consistently, the full (co)chaperone transcriptome and proteome data subsets were found to be significantly discordant (Mann-Whitney *P* value [*P*_MW_] = 5.0E−3). To identify concordance within the (co)chaperone data more precisely, the chaperone and cochaperone data were analyzed separately. The chaperones were not concordantly affected at the transcript level, the cochaperones were concordantly upregulated at the transcript level, and the (co)chaperones were concordantly downregulated at the protein level (see Fig. S8B in Text S1). This finding is consistent with numerous reports that the microbiota causes upregulation of host intestinal chaperone protein abundance, principally in the epithelium, and that the level of upregulation is typically highest in the cells that are closest to the microbiota (43, 44). The innate immune response is known to cause upregulation of heat shock protein gene expression, at least partially as a consequence of cross talk between the innate immune response and the unfolded protein response (43, 45).

All but 1 of the 15 (co)chaperones downregulated at the proteome level were not affected at the transcriptome level (Fig. 4D). This may have resulted from the microbiota affecting (co)chaperone posttranscriptional regulation, protein secretion, and/or protein degradation. Cellular export of chaperones has been reported, though the mechanism is unclear (46, 47), and it is possible that the microbiota somehow downregulates (co)chaperone export from cells of the mouse ileum, resulting in the observed discordancy. Some chaperones are known to associate with the E3 ubiquitin ligases that typically target unfolded/misfolded client proteins, resulting in their degradation (48).

We noted that some (co)chaperones were upregulated in the GF ilea in the transcriptome data subset and were unaffected in the proteome data subset. It is possible that transcription, translation, and protein degradation of these (co)chaperones were upregulated in the GF mice, resulting in increased abundance in the transcriptome and no significant net change in the proteome. We also noted that Stub1 itself was upregulated in the transcriptome data subset but was unaffected in the proteome data subset. STUB1 is known to autopolyubiquitinate itself (49), which may result in its degradation, and this may explain the discordancy that we observed. Intriguingly, PARK2 has a key role in derepressing translation of OxP transcripts, and it is also a chaperone-associated E3 ubiquitin ligase that typically targets unfolded/misfolded client proteins, resulting in their degradation (48). It is possible that PARK2 has a dual role in the host response to the microbiota, linking derepression of translation with chaperone-associated ubiquitination.

A summary of the ileum data set results is provided as Fig. 4E.

### Adaptation of the intestinal transcriptome to the microbiota varied significantly across globally diverse murine and microbiota populations.

A meta-analysis was performed to discover host genes that were affected by the microbiota (Fig. 1A). The meta-analysis used the microarray data originating from a globally diverse set of samples that varied in microbiota population, host strain, host sex, tissue location within the intestine, and sample preparation protocol (Table S8). The abundances of 2,132 genes were statistically significantly affected by germ status (Table S9). This is the first report of such an analysis. Ideally, a corresponding meta-analysis using proteomics data would also have been performed, but our proteomics comparison of conventional and germfree mouse intestinal tissue is the first of its kind.

10.1128/mSystems.00107-17.9 TABLE S8  OMiCC meta-analysis samples. Download TABLE S8, XLSX file, 0.02 MB. Copyright © 2017 Manes et al. 2017 Manes et al. This content is distributed under the terms of the Creative Commons Attribution 4.0 International license.

10.1128/mSystems.00107-17.10 TABLE S9  OMiCC meta-analysis data set. Download TABLE S9, XLSX file, 1.9 MB. Copyright © 2017 Manes et al. 2017 Manes et al. This content is distributed under the terms of the Creative Commons Attribution 4.0 International license.

**FIG 5:**
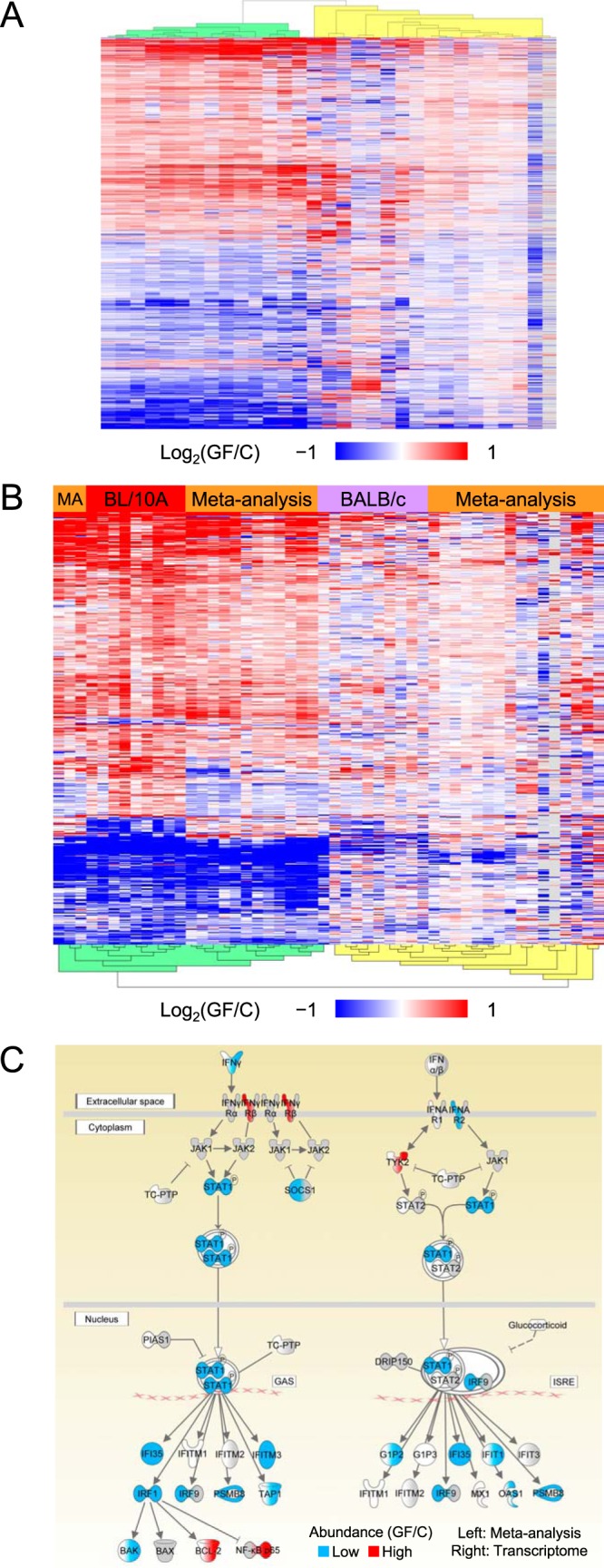
The transcriptome data subset and meta-analysis data set were partially concordant. (A) An HCA of the meta-analysis data set resulted in clustering of relatively strongly and weakly affected mice (indicated by the green and yellow dendrogram highlighting, respectively). Each row depicts a significantly affected gene (*n* = 2,132), and each column depicts an intrastudy pair of groups of mice (a “comparison group pair” consisting of a conventional group and a GF group) (both rows and columns were clustered; gray = missing value). (B) An HCA of the intersection of the transcriptome data subset and the meta-analysis (MA) data set resulted in clustering of strongly and weakly affected mice (indicated by the green and yellow dendrogram highlighting, respectively). Each row depicts a significantly affected gene (*n* = 530), each transcriptome column depicts a pair of mice (1 GF and one conventional from one microarray; sex and strain matched; red and lavender bars), and each meta-analysis column depicts an intrastudy “comparison group pair” (orange bars) (*q*_min_ [germ status] < 0.1 required for both sets of data [concordance of GF/C ratios was not required]; both rows and columns were clustered; gray = missing value). (C) The interferon signaling pathway was downregulated in the GF mice. Each pathway node was dually colored using GF/C ratios from the sets of meta-analysis (left) and transcriptome (right) data. The statistical significance data were as follows: *q*_e_ (transcriptome, downregulated) = 1.6E−6, *q*_e_ (proteome, downregulated) = 4.6E−2, *q*_e_ (meta-analysis, downregulated) = 1.7E−3, *P*_c _[meta-analysis] = 3.9E−2. The transcriptome node-level intraset concordancy was not statistically significant, but it was close (*P*_c_ = 5.7E−2; significance level = 0.05).

An HCA of the meta-analysis data partitioned the rows into two clusters of roughly equal sizes: one of upregulated genes and the other of downregulated genes (Fig. 5A). The HCA also partitioned the columns (depicting comparison group pairs) into two clusters. Germ status had a relatively strong effect on the leftmost cluster (14 columns; dendrogram highlighted green in Fig. 5A). This cluster contained mice from two studies: male C57BL/6J mice (jejunum, ileum, and colon; conventionalized for 4, 8, 16, and 30 days; GEO dataset accession no. GSE32513), and female C3H/HeN mice (ileum, conventional and conventionalized using a mouse microbiota for 8 and 60 days; GEO dataset accession no. GSE18056). From left to right, the columns of the green cluster Fig. 5A represent 11 GSE32513 columns (3 colon columns followed by 4 ileum columns and 4 jejunum columns) followed by 3 GSE18056 columns. The colon 30-day GSE32513 column data were aberrantly absent from the green cluster.

In comparison to the green cluster, germ status had a relatively weak effect on the other cluster (17 columns; dendrogram highlighted in yellow in Fig. 5A). It is likely that six columns of the yellow cluster were weakly affected because they originated from comparisons of GF versus conventionalized mice at early time points (days 1 and 2; all from the GSE32513 study). There was no obvious reason for the relatively weak effect shown in the other 11 columns. However, two columns of the yellow cluster (columns 4 and 5, counting from the left) represented data from mice that had been conventionalized using a human microbiota (GSE18056 study; 8 and 60 days, respectively), and it is possible that human-mouse microbiota differences somehow caused the relatively weak effect. We noticed that one column of the yellow cluster (column 7, counting from the left) was the colon 30-day GSE32513 column mentioned above and that the bottom half of this column roughly matched the corresponding data in the green cluster. In summary, the meta-analysis revealed that the mice were affected by germ status at two levels: a relatively low level and a high level. It is possible that this dissimilarity was due to the different mouse strains and/or different microbiota populations examined. The pattern of relatively strong and weak effects in the meta-analysis data set was consistent with the strain-dependent patterns seen in both the transcriptome and proteome data subsets (Fig. 1B and C).

An HCA of the significantly affected genes in the transcriptome data subset and the meta-analysis data set was performed (Fig. 5B), and the row and column partitioning was similar to that described above (Fig. 1B and C, 2, and 5A). With a single exception (the aberrant colon 30-day GSE32513 column), the strong/weak partitioning of the meta-analysis columns was the same in Fig. 5A and B. Similarly, the strong/weak partitioning of the transcriptome columns was the same in Fig. 1B and 5B except for a single BALB/c column. A correlation analysis was performed, and the correlation was highly significant (see Fig. S9A in Text S1). Reassuringly, the concordant changes between the transcriptome data subset and meta-analysis data set validated each other. The overall concordance across the transcriptome, proteome, and meta-analysis sets of data were examined (see Fig. S9B in Text S1). A significantly affected gene in one set was most likely to be unaffected or unquantified within the other two sets. However, if a gene was significantly affected within multiple sets, the effects were more likely to be concordant than discordant.

### Adaptation of the intestine to the microbiota affects hundreds of biological pathways.

Three hundred eighty-four Ingenuity pathways were significantly enriched in the meta-analysis data set (Table 1). Two hundred sixty-eight (70%) of the pathways were not enriched in the transcriptome or proteome data subsets (28 were downregulated, 60 were upregulated, and 180 were intraset discordant). Thus, the meta-analysis greatly broadened the discovery of pathways affected by germ status. As with the ileum data set, some of these pathways were downregulated immune system pathways and upregulated metabolic pathways, but the pathways varied greatly overall.

Twenty-eight pathways were downregulated in both the transcriptome and meta-analysis sets of data, and almost all were immune system pathways such as the interferon signaling pathway (Fig. 5C). Only three pathways, the glutathione-mediated detoxification pathway, the serotonin degradation pathway, and the xenobiotic metabolism signaling pathway, were upregulated in both the transcriptome analysis and meta-analysis, and these three were also upregulated in the proteome. Ninety pathways were downregulated in the concordant[T,M] gene set, and most were immune system pathways. Twenty-one pathways were upregulated in the concordant[T,M] gene set, and almost all were metabolism pathways.

Fifteen pathways were downregulated in the sets of transcriptome, proteome, and meta-analysis data, and almost all were immune system pathways such as the antigen presentation pathway (Fig. 3A) and the interferon signaling pathway (Fig. 5C). As mentioned above, three pathways were upregulated in all three sets of data. Eight pathways were downregulated in the concordant[T,P,M] gene set, and almost all were immune system pathways. Twenty-eight pathways were upregulated in the concordant[T,P,M] gene set, and almost all were metabolism pathways. Notably, the aryl hydrocarbon receptor signaling pathway was significantly enriched and intraset discordant in all three sets of data.

**FIG 6:**
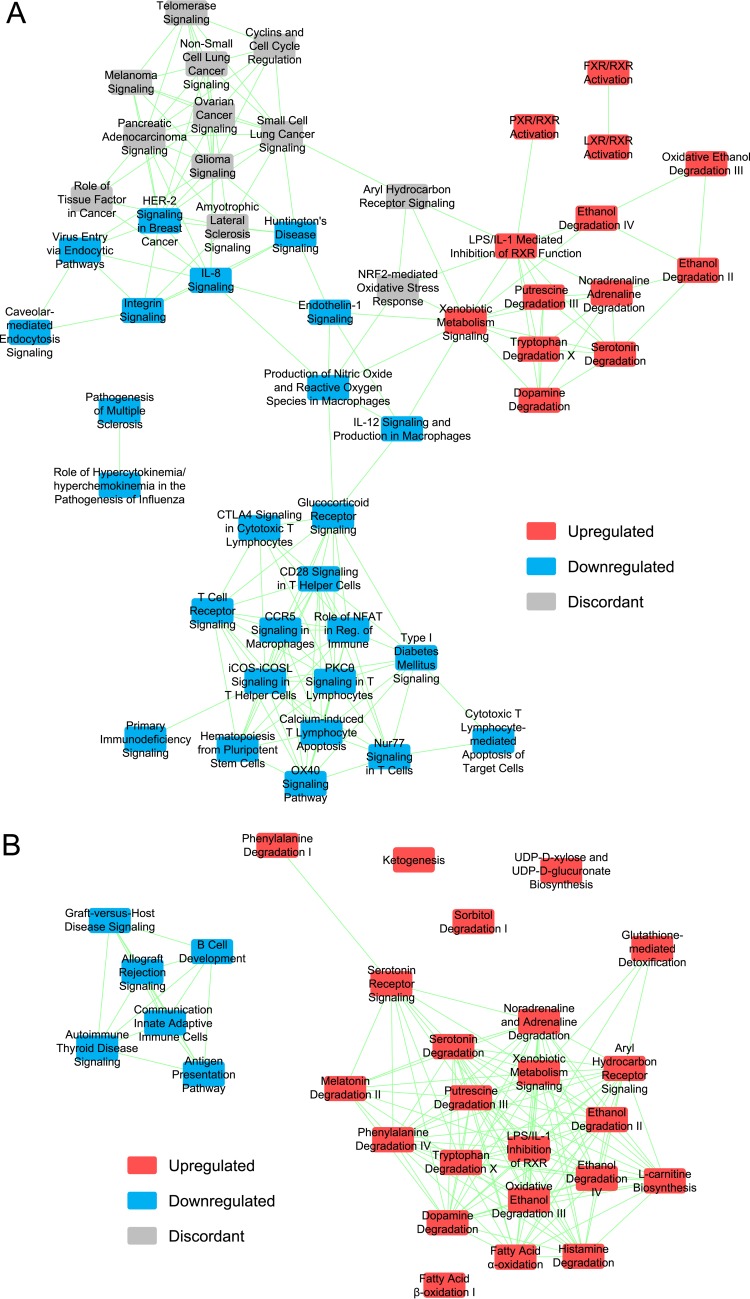
Genes affected concordantly within the ileum and meta-analysis data sets formed biofunctional clusters of overlapping pathways. Each node depicts a significantly enriched biological pathway, and each edge depicts overlapping genes that were significantly affected by germ status. Pathways were classified as upregulated if the annotation enrichment was found to be maximally significant by analyzing only the upregulated genes and as downregulated if the annotation enrichment was found to be maximally significant by analyzing only the downregulated genes. (A) The concordant[T,M] network (≥4 overlapping genes per edge) formed three clusters related to cancer (top left), metabolism (top right), and the immune system (top left and bottom center). (B) The concordant[T,P,M] network (with at least one instance of overlapping genes per edge) formed two independent clusters, one composed of downregulated immune system pathways (left) and the other of upregulated metabolic pathways (right).

Network analysis of the concordant[T,M] gene set resulted in three clusters (Fig. 6A). The top-right cluster in the figure panel primarily consists of upregulated metabolic pathways, the bottom-center cluster primarily consists of downregulated immune system pathways, and the third cluster (upper left) partially consists of downregulated immune system pathways. Interestingly, the upper half of the third cluster was principally composed of intraset discordant cancer pathways. In a related result, the colorectal cancer metastasis pathway was enriched and intraset discordant in the meta-analysis data set (*q*_e _[up + down] = 1.6E−12). This pathway was not enriched in either the transcriptome data subset or the proteome data subset. Thus, the microbiota affected numerous cancer pathways at the transcript level. Network analysis of the concordant[T,P,M] gene set resulted in two clusters (Fig. 6B). One was principally composed of downregulated immune system pathways, and the other was principally composed of upregulated metabolic pathways. This network might constitute the core set of pathways that are most broadly and strongly affected by the microbiota.

### Conclusions.

The microbiota is an important factor for human health and biomedical research, and GF animals are a valuable tool for discovering microbiota effects on the host. A hypothetical model of some of the results is summarized in Fig. S10 in Text S1, and compendia of illustrations of the affected pathways have been prepared and are available upon request. So far, most system-level studies comparing conventional and GF animals have focused on transcriptomic analyses. Although it has been found that protein abundance under steady-state conditions is primarily determined by transcript abundance, the spatial and temporal variations of mRNAs, the local availability of resources for protein biosynthesis, posttranscriptional regulation, and targeted protein degradation strongly influence the relationship between protein levels and their coding transcripts. Transcript fluctuations can be buffered at the level of protein concentration; consequently, analysis of transcript levels alone is generally not sufficient to predict protein levels in many scenarios (50), including during host-microbiota interactions. To address this, we performed the first proteomic comparison of the gastrointestinal tract of conventionally raised and GF mice alongside a corresponding transcriptomic analysis. Multiple gene products and hundreds of biological pathways were found to be affected by germ status in the mouse terminal ileum, and protein-level variance often did not reflect a similar change in the corresponding transcript level.

While transcriptomics analysis of small samples (e.g., from laser capture microdissection) is now routine, proteomics technology needs to continue to be researched and developed to enable deep analyses of small-mass samples. When such analyses become possible, this work will provide the foundation for the studies of cell-specific changes in the proteome caused by the microbiota. Now, on the basis of our analysis and our previous work, we can speculate that some of the changes that we observed at the proteome and transcriptome levels are caused by changes in the abundance of specific cell populations in the ileal tissue. Our previous study (14) had shown enrichment of T and B lymphocytes caused by the microbiota. However, this type of analysis does not discriminate between subpopulations, which would be more interesting to analyze. Therefore, future studies using single-cell transcriptome sequencing (RNA-seq) would enable addressing this issue.

Our report demonstrates that transcript abundance cannot always reliably predict protein abundance differences caused by the perturbations of the microbiota; provides a resource to study the effect of the microbiota on the host at the functional, protein, and pathway levels; and reinforces the idea of the importance of measuring protein abundance alterations to reveal molecular mechanisms behind the phenotypic changes caused by changes in the microbiota.

## MATERIALS AND METHODS

### Mouse ilea.

This study was approved by the NIH Animal Care and Use Committee. Rederived GF and conventional mice (female and male; BALB/c and C57BL/10A strains; 6 to 8 months old) were prepared by Taconic Biosciences, Inc. (Hudson, NY). The conventional mice were housed under normal conditions and maintained using standard procedures. Six GF mice were prepared for each experimental class (two sexes, two strains, 24 GF mice in total) with the goal of having 5 mice per experimental class (two germ statuses, two sexes, two strains, 40 mice in total). Samples (e.g., feces) from the GF mice were analyzed to ensure the complete absence of any microbial contamination, and mice were discarded if any sample tested positive. This resulted in only 4 GF female C57BL/10A ilea being available. The ilea were harvested, placed into and gently flushed with ice-cold phosphate-buffered saline (PBS) to wash away microbes, segmented, and immediately frozen on dry ice. One set of the segments was used for the transcriptomics experiments, and another set was used for the proteomics experiments. This resulted in the production of data referred to here as the “ileum data set,” consisting of the transcriptome and proteome “data subsets.”

### Transcriptomics.

Total RNA was isolated from the ilea using Total RNeasy minikits (Qiagen, Hilden, Germany). Fluorescently (green and red) labeled cDNA was prepared using 20 μg of total RNA and 2 μl of 1 mM Cy3-dUTP and Cy5-dUTP, respectively (GE Healthcare, Chicago, IL), 1 μg of oligo(dT)_12–18_ primer, 0.5 mM dATP, 0.5 mM dCTP, 0.5 mM dGTP, 0.15 mM dTTP, 10 mM dithiothreitol, 1.5 μl of 200 U/μl SuperScript II reverse transcriptase, and 0.5 μl of 20 U/μl RNase inhibitor (all from Thermo Fisher Scientific Inc., Waltham, MA, unless noted otherwise). The resulting cDNA was purified using Vivaspin 500 columns (Sartorius AG, Göttingen, Germany) (10,000 molecular weight cutoff [MWCO]). Each sample pair (red and green; 38 samples in total) was cohybridized to a gene microarray (Agilent Technologies, Santa Clara, CA) (45,220 spots, 37,558 reporter spots, 35,958 reporter probes, 60-mer probes, “4 by 44 k” slide format, design number 017138) using a MicroArray User Interface (MAUI) hybridization system (BioMicro Systems Inc., Salt Lake City, UT). A reference-free experimental design was used. Each microarray was used to analyze 1 GF-mouse sample and one conventional-mouse sample (sex and strain matched). To mitigate label effects, half of the samples of each experimental class were randomly selected to be labeled green and the other half red. The dual-color microarrays were scanned using a Microarray Scanner System, and the resulting images were processed using Feature Extraction (both Agilent Technologies). These data are available at the Gene Expression Omnibus data repository at the National Center for Biotechnology Information (GEO Platform GPL9354; GEO entry identifier GSE95650; http://www.ncbi.nlm.nih.gov/geo/).

Gene symbols were retrieved from bioDBnet (12 March 2016; https://biodbnet-abcc.ncifcrf.gov/) (51) using the gene and transcript identifiers within the GEO Platform data set. This resulted in 22,861 mouse gene symbols being associated with 29,819 reporter probes. Each reporter spot foreground and background intensity value represented the corresponding median pixel intensity value. Each spot background intensity value was set to be a maximum of four times the median background intensity value (calculated across each microarray and color channel; this affected 251 of the 1,427,204 background intensity values). For each ileum sample and spot, if the net intensity (foreground − background) value was <10, it was replaced with a random number between 10 and 20 (this range was roughly consistent with the range of the background values, as the median background intensity value was 35.5; this affected 31,139 of the 1,427,204 net intensity values). For each microarray/color channel/probe, the arithmetic mean of the net intensity values was calculated across replicate spots. The resulting values were log_2_ transformed.

A statistical analysis was performed using BRB-ArrayTools (http://brb.nci.nih.gov/BRB-ArrayTools/) (52) to identify probe intensity values that were significantly affected by germ status. To this end, intra-array LOWESS normalizations (span = 0.4) were performed. The resulting data were used for analyses of variance (ANOVAs) that used a statistical model designed specifically for reference-free dually labeled microarray data (53). The BRB-ArrayTools “Random Variance Model” option was used. The ANOVA model estimated array effects as well as the effects due to the eight experimental classes (two germ statuses, two sexes, two strains). The resulting data were used for *post hoc* pairwise comparison testing across the experimental classes (35,958 probes × 28 pairs of experimental classes = 1,006,824 tests), which resulted in a *q* value (i.e., a false-discovery-rate value) for each test. For each probe, the minimum *q* value (*q*_min_) was calculated across the four relevant pairs of experimental classes (GF mice versus conventional mice, sex and strain matched). The data were filtered by requiring *q*_min_ values to be <0.1. This resulted in 3,817 probes (10.6%) passing this filter, and this corresponded to 3,063 genes (13.4%). Pearson and Spearman correlation *P* values were calculated using 2-tailed *t* tests. HCAs were performed using Genesis (complete linkage clustering using Euclidean distance) (54). For each probe, the arithmetic mean of the log_2_-transformed and LOWESS-normalized GF/C (germfree/conventional) ratios (described above) was calculated across the microarrays, and the anti-log_2_ values determined were used as the GF/C values for the pathway analyses.

An additional statistical analysis was performed using InfernoRDN (https://omics.pnl.gov/software/infernordn) (55) to identify probe intensity values that were significantly affected by sex and/or strain. To this end, an interarray LOESS (locally weighted scatterplot smoothing) normalization (reference = median value, span = 0.4) was performed. The resulting data were used to perform a 3-way ANOVA (germ status, sex, and strain; an array effect was not included) for each probe. Each 3-way ANOVA produced a *q* value for each of the three effects (germ status, sex, and strain) and for each of the four interactions (germ status plus sex, germ status plus strain, sex plus strain, and germ status plus sex plus strain). For each probe, a minimum *q* value (*q*_min_) was calculated for each experimental parameter (germ status, sex, and strain). For example, *q*_min_(sex) equaled the minimum of the *q* values that involved the sex parameter [*q*(sex), *q*(germ status plus sex), *q*(sex plus strain), and *q*(germ status plus sex plus strain)]. The data were filtered by requiring *q*_min_ values to be <0.1. This resulted in 1,180 (3.28%), 574 (1.60%), and 22,758 (63.3%) probes passing the germ status, sex, and strain filters, respectively, and this corresponded to 917 (4.01%), 462 (2.02%), and 15,784 (69.0%) genes, respectively. HCAs were performed using Genesis as described above. Note that these data were not used for pathway analyses.

### Proteomics.

The ileum samples were blocked and randomized to mitigate any potential systematic biases present during sample preparation (56). Each sample was homogenized in 500 µl of lysis buffer (freshly prepared 100 mM HEPES-NaOH [pH 8], 8 M urea, 10 µM bestatin hydrochloride, 10 µM pepstatin A, PhosStop phosphatase inhibitor cocktail [F. Hoffmann-La Roche Ltd., Basel, Switzerland] [1 tablet/10 ml]). Homogenization was performed using an Omni TH tissue homogenizer equipped with 7-mm-by-110-mm “hard tissue” disposable plastic tips (Omni International, Kennesaw, GA) (25% to 50% setting for 60 s). The homogenates were bath sonicated for 10 min at room temperature, and bicinchoninic acid protein concentration assays (Thermo Fisher Scientific Inc.) were performed.

Dithiothreitol was added to 500 µg (protein mass) of each ileum homogenate, and the samples were incubated at 60°C for 45 min to reduce protein cysteine residues (final dithiothreitol concentration = 10 mM). Iodoacetamide was added to each sample (final concentration = 50 mM), and the samples were incubated at room temperature for 20 min in darkness to alkylate cysteine residues. Each sample was diluted using 100 mM HEPES-NaOH (pH 8) such that the final urea concentration was 1 M. Protein digestion was performed by adding sequencing-grade modified trypsin (Promega Corp., Madison, WI) (1:100 [wt:wt] protein trypsin/sample) and incubating the samples at 37°C for 19 h. Each sample was acidified by adding formic acid (final concentration = 1% [vol/vol]), and solid-phase extraction was performed using Sep-Pak C_18_ columns (Waters Corp., Milford, MA).

A reference “pool” sample for relative quantifications was prepared by pooling 5 µl (50 µg) of all 39 ileum samples. Six sets of iTRAQ 8-plex reagent (AB Sciex Pte. Ltd., Framingham, MA) were used to label the samples following the manufacturer’s instructions (100 µg of peptide mass per labeling reaction; ≥1 reference sample per iTRAQ 8-plex set). The resulting six samples underwent TiO_2_ phosphopeptide enrichment as described previously (57). The resulting flowthrough samples (i.e., the nonphosphorylated peptides used in this study) were fractionated using strong cation exchange high-pressure liquid chromatography (HPLC) and a 2.1-mm-by-200-mm PolySULFOETHYL A column (Poly LC Inc., Columbia, MD) (5-µm diameter; 20-nm pore size; mobile phase A = 10 mM KH_2_PO_4_-HCl [pH 2.8] with 25% [vol/vol] acetonitrile; mobile phase B = mobile phase A with 1 M KCl; 30 [run 1] or 33 [runs 2 to 6] fractions per run). The fractions were subjected to solid-phase extraction using ZipTip C_18_ tips (Millipore Corp., Billerica, MA).

The samples were analyzed using a Nano-LC Ultra two-dimensional (2D) HPLC system (Eksigent Technologies [acquired by AB Sciex Pte. Ltd.]) coupled via nano-electrospray (nano-ES) to a linear trap quadrupole (LTQ) Orbitrap Velos mass spectrometer (Thermo Fisher Scientific Inc.). The LC system was equipped with a trap column (8-cm length, 100 µm inner diameter [i.d.]) and a packed-tip column (15-cm length, 50 µm i.d.), and both were packed with Halo ES C_18_ resin (Michrom Bioresources, Inc. [acquired by Bruker Corp., Billerica, MA]) (2.7-µm diameter, 16-nm pore size). Each LC-tandem mass spectrometry (LC-MS/MS) run included a 120-min linear gradient (3% to 35% mobile phase B), a 10-min column regeneration step (90% mobile phase B), and a 30-min column reequilibration step (3% mobile phase B) (300 nl/min flow rate; mobile phase A = 0.1% [vol/vol] formic acid–H_2_O; mobile phase B = 0.1% [vol/vol] formic acid–acetonitrile). The top 6 most intense ions per precursor ion scan were selected for MS/MS (both collision-induced dissociation [CID] and higher-energy collisional dissociation [HCD]). This resulted in 236 LC-MS/MS runs and 4,434,402 MS/MS spectra (not including runs of blanks and quality control [QC] standards).

The resulting spectra were analyzed using Proteome Discoverer v2.0.0.802 (Thermo Fisher Scientific Inc.). The spectra were searched against the UniProt protein sequence database (taxonomy = *Mus musculus*; 84,602 protein sequences; UniProt release 2015_01; 7 January 2015; http://www.uniprot.org/) (58) supplemented with the sequences of protein standards and common contaminants (416 protein sequences). The spectra were searched using both Sequest HT (a component of Proteome Discoverer) (59) and Mascot Server v2.5.1.0 (Matrix Science, Inc., Boston, MA) (60). The search settings included the following: fully tryptic, ≤2 missed cleavages, and 20 ppm precursor mass tolerance. The fragment mass tolerance settings were 0.8 Da (CID; Mascot), 1 Da (CID; Sequest HT), 0.02 Da (HCD; Mascot), and 0.06 Da (HCD; Sequest HT). The search settings included one static modification: carbamidomethylation (Cys). The search settings included seven dynamic modifications: deamidation (Asn, Gln), oxidation (Met), iTRAQ8plex (Lys, Tyr, peptide N termini), acetylation (Lys, peptide N termini), amidation (protein C termini), Met loss (protein N-terminal Met), and Met loss plus acetylation (protein N-terminal Met).

Percolator (a component of Proteome Discoverer) was used to analyze all of the resulting peptide-spectrum matches (PSMs), and the PSMs were required to have Percolator *q* values of <0.01 (61, 62). This resulted in 577,567 PSMs with a “refined” false-discovery rate (63) of 0.0046. This corresponded to 29,954 peptide groups (ignoring peptide modifications, this corresponded to 22,573 peptides). The data were further filtered by requiring each protein group identification to correspond to either ≥2 peptide groups and ≥4 PSMs or ≥10 PSMs. This resulted in 3,088 protein group identifications (not including protein standards and common contaminants; false-discovery rate = 0.018), which corresponded to 2,968 genes (gene symbols were retrieved from bioDBnet as described above). Of the 3,088 protein group identifications, 2,896 (93.8%) corresponded to ≥2 peptide groups, and 2,823 (91.4%) corresponded to ≥2 peptides (ignoring peptide modifications).

Proteome Discoverer was used to perform iTRAQ 8plex quantification relative to the quantification reference “pool” sample described above. Each protein group abundance value was equal to the median of the corresponding PSM sample/pool ratios. Proteome Discoverer was used to perform an abundance bias correction (a central-tendency normalization) using the median protein group abundance values. The resulting 39 sample normalization values (1 per ileum) ranged from 0.52 to 1.87. The geometric mean of the 39 "log-absolute values" was 1.216 (if a normalization value was >1 or <1, then the corresponding log-absolute value was defined as the normalization value or the reciprocal normalization value, respectively), which indicated that the mean bias was 21.6%. Six subsets of the set of 39 normalization values were each defined by association with one of the six experimental parameters (conventional, GF, female, male, C57BL/10A, and BALB/c), and the geometric mean of each subset of normalization values was nearly unity (0.98, 0.96, 1.02, 0.92, 0.93, and 1.01, respectively), which indicated that the biases were largely independent of all six experimental parameters. The raw mass spectra and the Proteome Discoverer data were deposited in the ProteomeXchange Consortium database (http://proteomecentral.proteomexchange.org) via the PRIDE partner repository (64) with data set identifier PXD003177.

The protein group abundance values were log_2_ transformed, and InfernoRDN (https://omics.pnl.gov/software/infernordn) (55) was used to perform 3-way ANOVAs. As with the transcriptomics InfernoRDN 3-way ANOVAs (described above), each ANOVA produced a *q* value for each effect (germ status, sex, and strain) and interaction, and the data were filtered by requiring the minimum *q* value (*q*_min_, calculated for each experimental parameter) to be <0.1. This resulted in 242 (7.84%), 13 (0.42%), and 539 (17.5%) protein groups passing the germ status, sex, and strain filters, respectively, and this corresponded to 219 (7.38%), 12 (0.40%), and 495 (16.7%) genes, respectively. HCAs were performed using Genesis as described above. For each protein group, the geometric mean of the protein group abundance values (normalized; not log_2_ transformed) was calculated across the GF samples, the same was calculated for the conventional samples, and the ratios of the two geometric means were used as the GF/C values for the pathway analyses.

### Meta-analysis.

The meta-analysis used eight previously generated, publicly available gene microarray data sets described in 10 publications (12, 65–73). Throughout this article, these data are explicitly referred to as the “meta-analysis data set” (the “transcriptome data subset” exclusively refers to the mouse ileum experiments described above; the meta-analysis did not use any of the transcriptome data subset). All of the meta-analysis data were from experiments that used GF and conventional (including conventionalized) mice, and all of the analyses were of small-intestine or large-intestine whole-tissue samples (243 samples in total).

The data were downloaded from the Gene Expression Omnibus data repository at the National Center for Biotechnology Information (http://www.ncbi.nlm.nih.gov/geo/) (identifiers GSE1392, GSE5198, GSE8006, GSE18056, GSE23573, GSE32084, GSE32513, and GSE46952). The meta-analysis was performed using OMics Compendia Commons (OMiCC; https://omicc.niaid.nih.gov/) (74), a community-based online toolset for analyzing gene expression data. The results of the meta-analysis are available online (https://omicc.niaid.nih.gov/myProject/showResult/1118?ac=0SW9E3XLH). In OMiCC, the samples were arranged into 43 intrastudy “sample groups” (groups of biological replicates; minimum of three replicates per group), and intrastudy pairs of the sample groups (each pair contained one GF group and one conventional group) were arranged into 31 “comparison group pairs.” The meta-analysis tested for gene expression that was significantly affected by germ status (using only intrastudy comparisons), and it produced two *q* values for each gene [OMiCC meta-analyses always produce a *q*(up) value and a *q*(down) value for each gene]. For each gene, the minimum of the two *q* values was calculated (*q*_min_), and the data were filtered by requiring *q*_min_ values of <0.1 (2,132 genes passed this filter). HCAs were performed using Genesis as described above. For each gene, the geometric mean of the GF/C (germfree/conventional) ratios was calculated across the “comparison group pairs,” and these values were used as the GF/C values for the pathway analyses.

### Pathway analyses.

Ingenuity pathway analysis (Qiagen) was used to perform “Core Analyses” to discover biological pathways that were significantly affected by germ status. Each Ingenuity analysis was performed using sets of genes that were significantly affected by germ status and categorized as follows: “meta-analysis,” “transcriptome,” “proteome,” “concordant[T,P,M],” “concordant[T,P],” “concordant[T,M],” “discordant[T≠P],” “discordant[T\P],” “Discordant[P\T],” and “invariant[P].” For each of these 10 gene sets, two additional gene subsets were analyzed; one subset consisted of the upregulated genes, and the other consisted of the downregulated genes (e.g., transcriptome[up], discordant[P\T, down], proteome = proteome[up + down]). All 30 IPAs used the same IPA version (June 2016 release; Application Build 389077m; Content version 27821452).

The meta-analysis, transcriptome, and proteome gene sets (2,132, 3,063, and 219 genes, respectively) each consisted of the genes that were significantly affected by germ status (*q*_min_ = <0.1, as described above). The concordant[T,P,M] gene set (33 genes) consisted of the intersection of the aforementioned transcriptome, proteome, and meta-analysis gene sets, and the 3 GF/C ratios (transcriptome, proteome, and meta-analysis) for each gene were required to be concordant (i.e., all 3 GF/C ratios = <1, or all three = >1). The concordant[T,P] gene set (88 genes) was the same except that only the transcriptome and proteome data subsets were considered. Likewise, the concordant[T,M] gene set (441 genes) was the same except that only the transcriptome and meta-analysis gene sets were considered.

Each gene in the discordant[T\P] gene set (583 genes) was significantly affected by germ status in the transcriptome data subset (*q*_min_ = <0.1), was confidently identified and quantified in the proteome data subset, and was not significantly affected by germ status in the proteome (*q*_min_ = ≥0.1) or else the transcriptome and proteome GF/C ratios were in opposite directions (i.e., one represented upregulation and the other downregulation). The discordant[P\T] gene set (118 genes) was the same but with the transcriptome and proteome data subsets inverted. The discordant[T≠P] gene set (696 genes) consisted of the union of the discordant[T\P] and discordant[P\T] sets. Lastly, the invariant[P] gene set (60 genes) consisted of the genes significantly affected at the proteome level by germ status (*q*_min_[germ status] = <0.1) and not significantly affected at the proteome level by sex and strain (*q*_min_[sex] = ≥0.1 and *q*_min_[strain] = ≥0.1).

Each Ingenuity analysis used one of the 30 sets of genes described above (10 “up + down” gene sets, 10 “up” gene subsets, and 10 “down” gene subsets). Each Ingenuity analysis also required a reference set of genes, and each was the corresponding unfiltered (by *q*_min_) experimental gene set (the whole-mouse genome was used for the meta-analysis analyses). For each Ingenuity analysis, a gene annotation enrichment *q* value (*q*_e_) was calculated for each gene annotation (e.g., representing a biological pathway or function) by using a right-tailed Fisher exact test and subsequently applying Benjamini-Hochberg multiple-hypothesis analysis. Note that this algorithm (the Fisher exact test plus Benjamini-Hochberg test) was itself indifferent to concordancy in the direction of the GF/C values. The definition of *q*_e_ is that it represents the estimated fraction of false-positive results within the discovered set of enriched annotations (the set defined by assuming that the *q*_e_ value represents the significance level).

The “up + down” analyses were used to discover gene annotations that were statistically significantly enriched within the set of gene products that were significantly affected by germ status. Likewise, the “up” analyses were used to discover annotations that were significantly enriched within the set of upregulated gene products and the “down” analyses were used to discover annotations that were significantly enriched within the set of downregulated gene products. For some of the annotations, the “up” or “down” analysis resulted in a *q*_e_ value that was lower (i.e., represented greater statistically significance) than the corresponding *q*_e_ value from the “up + down” analysis. Mathematically, this is because *q*_e_ is dependent on both the number of affected genes that are assigned the annotation and the number of affected genes that are not assigned the annotation. Consequently, compared to an “up + down” analysis, an “up” or “down” analysis can reduce either of these numbers, and this can result in an increase or decrease in the *q*_e_ value.

The sets of biological pathways were filtered by requiring *q*_e_ values of <0.1 (428 pathways passed this filter in at least 1 of the 30 analyses). Pathways were classified as “upregulated” if the annotation enrichment was maximally significant by analyzing the “up” gene subset (that is, *q*_e_[up] < *q*_e_[up + down] and *q*_e_[up] < *q*_e_[down] and *q*_e_[up] < 0.1), and likewise for the pathways classified as “downregulated.” This definition should not be confused with the biological activation or inactivation of the pathways, as a pathway may be inactivated due to the upregulation of specific genes/proteins, or, alternatively, a pathway can be activated due to the downregulation of specific genes/proteins. Pathways were classified as “intraset discordant” (not to be confused with interset discordance such as transcriptome-proteome discordance) if the annotation enrichment was maximally significant by analyzing the “up + down” gene set (that is, *q*_e_[up + down] ≤ *q*_e_[up] and *q*_e_[up + down] ≤ *q*_e_[down] and *q*_e_[up + down] < 0.1).

The pathway nodes colored red and blue were significantly (*q*_min_ = <0.1) increased and decreased (respectively) in abundance due to germ status. The pathway nodes colored gray were not significantly affected (*q*_min_ = ≥0.1), and the pathway nodes colored white represent those that were not experimentally observed. Note that OMiCC performs an internal data filtration step; consequently, some meta-analysis nodes are colored white even though the corresponding genes were present in the original microarray data sets. Dual shading of a pathway node either depicted data from multiple gene products from a single data set or depicted data from two data sets (e.g., transcriptome and proteome), as indicated. For each of the 30 analyses, Ingenuity analysis was used to produce five networks of overlapping biological pathways. Each node depicted a biological pathway, and each edge depicted ≥1 overlapping gene products that were significantly affected by germ status. The minimum number of significantly affected overlapping gene products ranged between one and five. The nodes were arranged using the Ingenuity “organic layout” algorithm. Intraset concordancy *P* values (*P*_c_) were calculated using two-tailed binomial tests (significance level = 0.05). Prism v7.02 (GraphPad Software, Inc., La Jolla, CA) was used to perform two-tailed Mann-Whitney tests (significance level = 0.05) to produce interset discordancy *P* values (*P*_MW_) and also to produce scatter dot plots.

Chaperone, cochaperone, and E3 ubiquitin ligase protein-protein interactions were retrieved from the STRING database (v10.0) (http://string-db.org/) (75). Only “database” interactions (from manually curated pathways) and “experimental” interactions were used to analyze protein-protein interactions. The STRING database assigns a confidence score to every interaction and classifies interactions as low, medium, high, and very high confidence based on score thresholds of 0.15, 0.4, 0.7, and 0.9, respectively. The set of (co)chaperones (76) and the set of chaperone-associated E3 ubiquitin ligases that target misfolded proteins (48) were previously reported. This set of interactions was manually supplemented with additional data (77–82). Cytoscape was used for network visualization (http://www.cytoscape.org/) (83). The STRING database was also used to produce transcript coexpression networks.

### Data availability.

We have deposited the required mass spectrometry proteomics data in the ProteomeXchange Consortium (http://proteomecentral.proteomexchange.org) via the PRIDE partner repository (ProteomeXchange submission title, “Germ-free mouse ileum iTRAQ shotgun LC-MSMS”) under ProteomeXchange accession number PXD003177. The microarray data have been deposited in NCBI's Gene Expression Omnibus and are accessible through GEO Series accession number GSE95650.
