# Supplementary material for: Multi-omics Comparative Analysis Reveals Multiple Layers of Host Signaling Pathway Regulation by the Gut Microbiota
Source: mSystems. 2017 Oct 24;2(5):e00107-17. doi: 10.1128/mSystems.00107-17 (PMC5655592; doi:10.1128/mSystems.00107-17)
Supplement: TEXT S1 [file sys005172143s1.docx]

A B


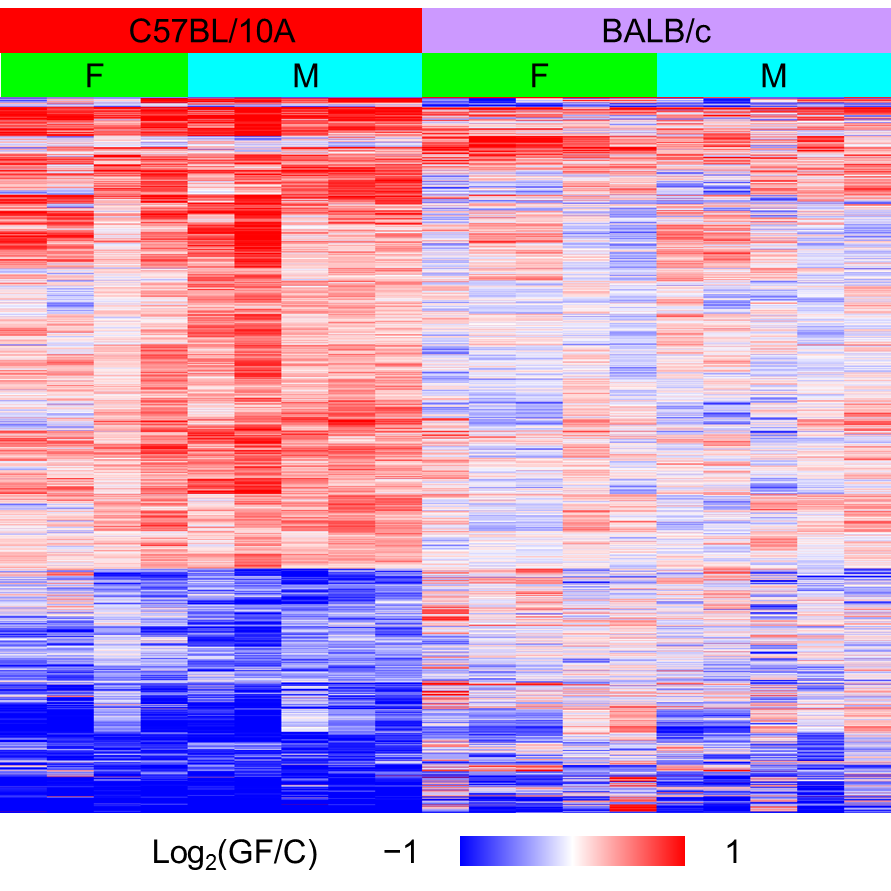

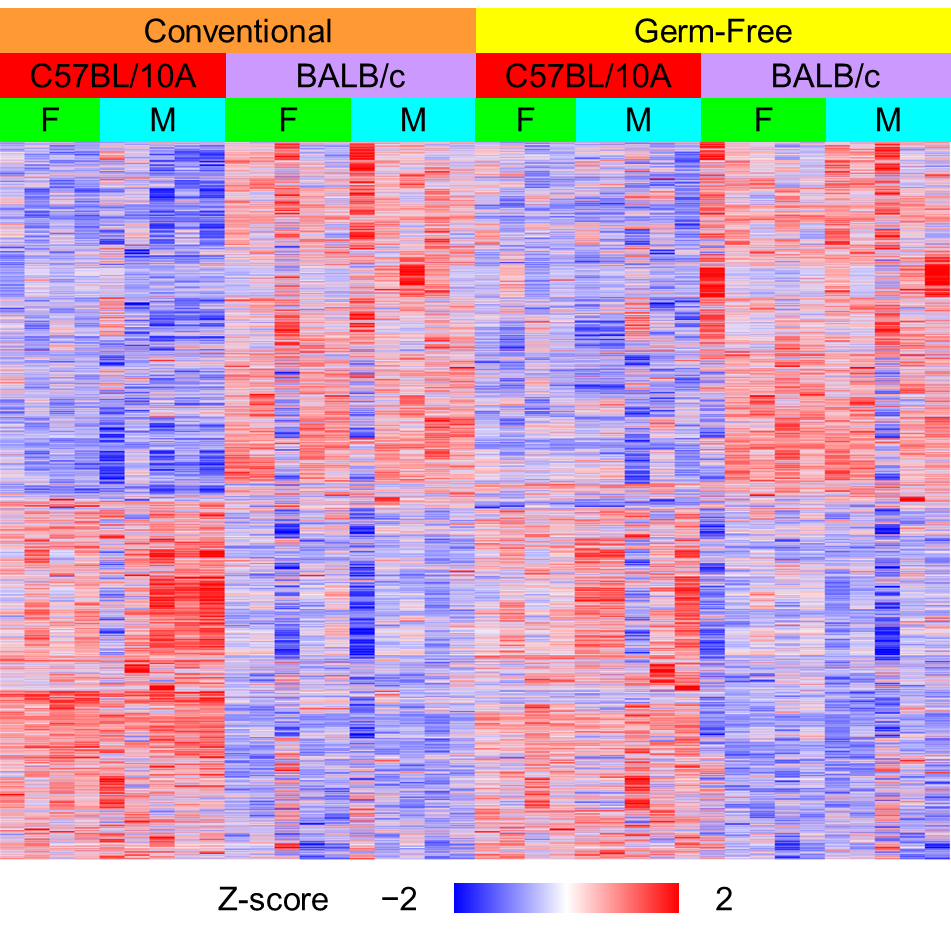


C D


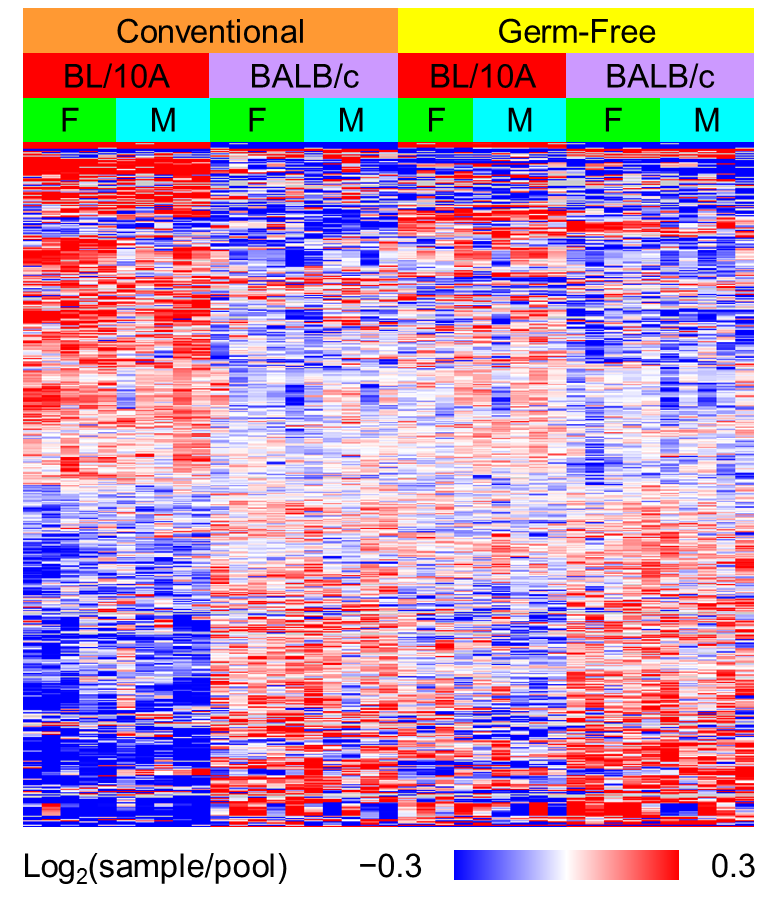

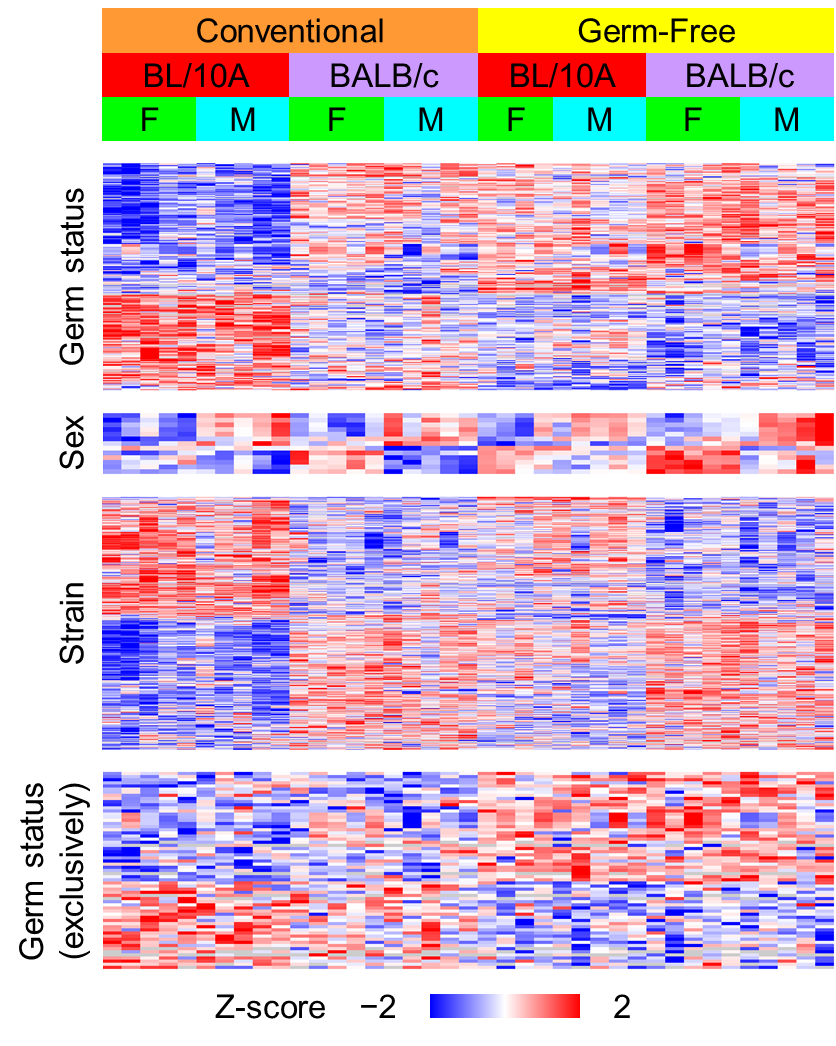


**Figure S1. A) HCA heatmap of the transcriptome data subset.** The same analysis as in Figure 1B except that the HCA was performed using Log_2_(GF/C) values instead of Log_2_(GF/C)/σ_st_ values. **B). HCA heatmap of the transcriptome data subset analyzed using InfernoRDN.**

Each column depicts a sample, and each row depicts a probe that was significantly affected by germ status, sex, and/or strain (n = 22,979; only rows were clustered; gray = missing value). The color-bar indicates the relative abundance of the probe: Z-score(LOESS(Log_2_(intensity))). **C).** **HCA heatmap of the proteome data subset.** Each column depicts an ileum, and each row depicts a protein group that was significantly affected by germ status, sex and/or strain (n = 609; only rows were clustered; gray = missing value). **D).** **HCA heatmaps of the proteome data subset for the experimental parameters**. Each column depicts an ileum, and each row depicts a protein group that was significantly affected by an experimental parameter (from top to bottom, n = 242, 13, 539, 63; only rows were clustered; gray = missing value). The color-bar indicates the relative protein group abundance: Z-Score(Log_2_(sample/pool)).


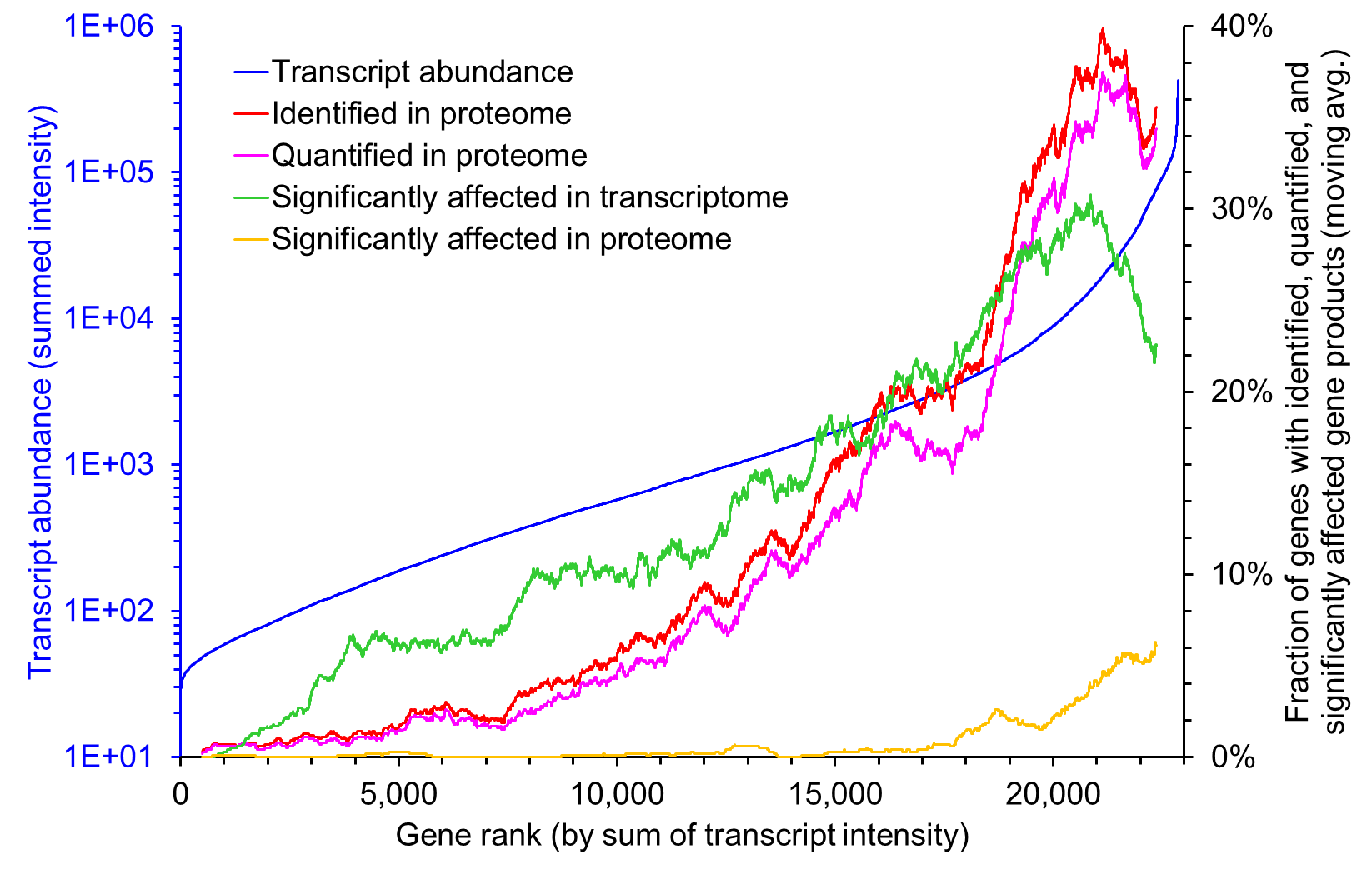


**Figure S2. Relationship between transcript abundance and the frequency of gene product identification, quantification, and significant change.**

For each gene, a transcript abundance value was calculated. Specifically, the InfernoRDN probe intensity values (log_2_-transformed and LOESS normalized) were anti-log_2_-transformed, the 90th percentile was calculated across the ileum samples, and the resulting values were summed across the probes. The genes were ranked by transcript abundance, and the frequency of gene product identification, quantification, and significant change (q_min_[germ status] < 0.1; the transcript q-values were from BRB-ArrayTools) was calculated (moving average with an interval of 1,000).

A


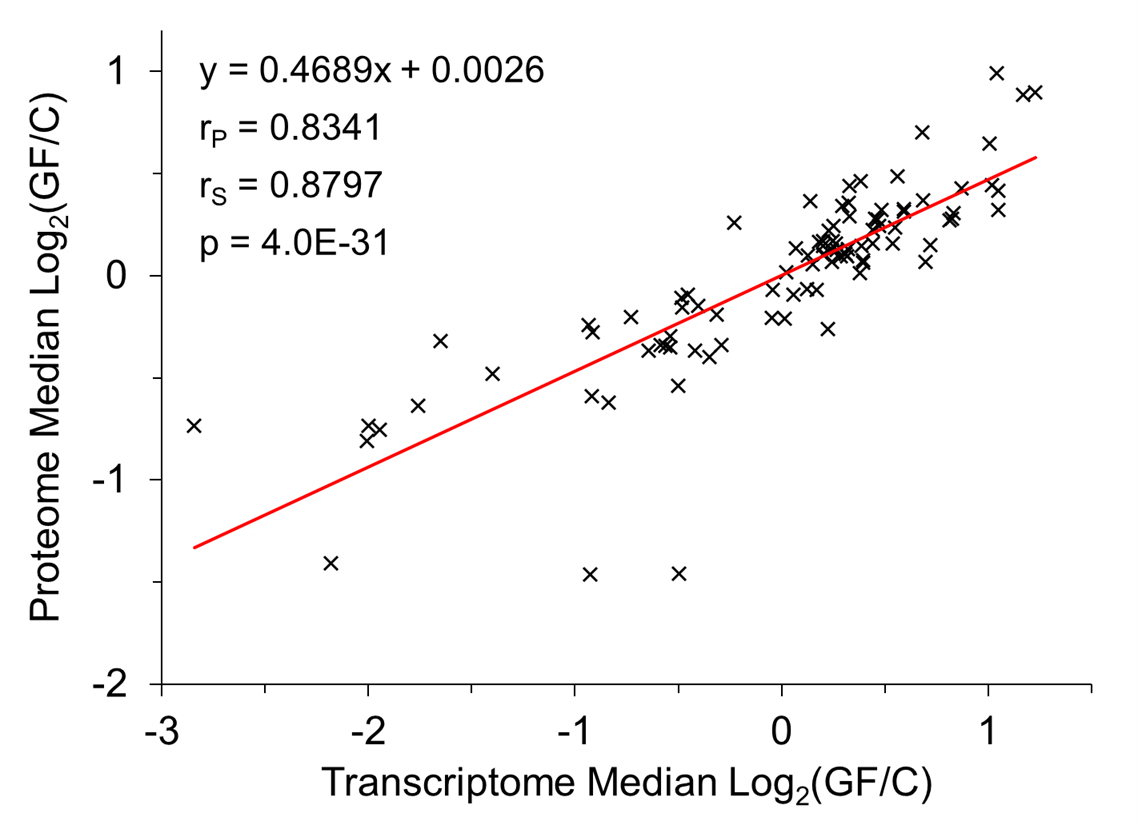


B


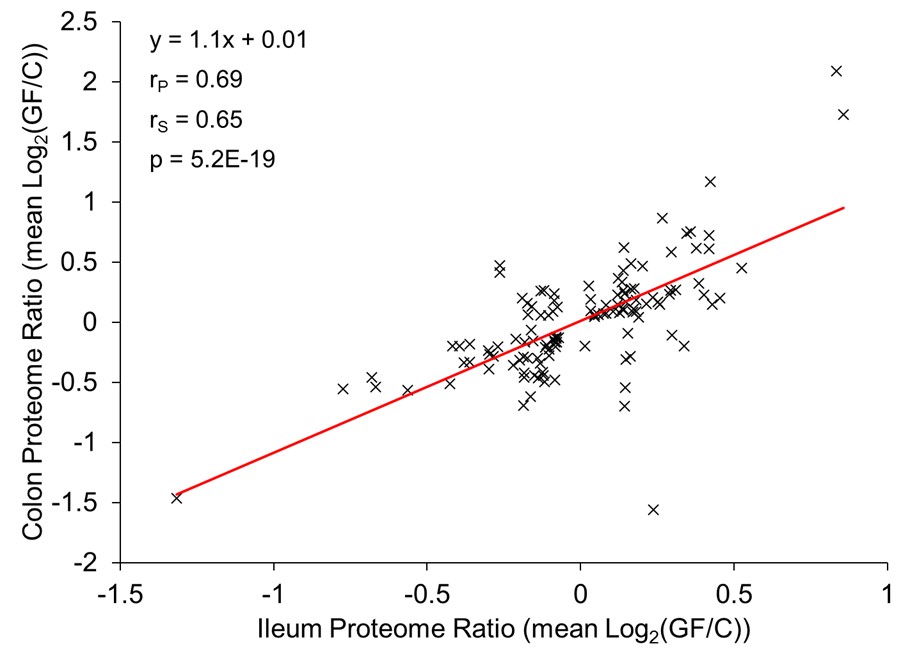


**Figure S3. A). Correlation of ileum and proximal colon protein abundances that were significantly affected by germ status.** The ileum proteome data subset from this study (q_min_[germ status] < 0.1) was compared at the gene level to a previously reported mouse colon quantitative shotgun proteomics dataset (ten male GF C57BL/6 mice; at 57 – 62 days old, five were conventionalized, and the other five were mock-conventionalized; proximal colons were harvested 14 days later; q[germ status] < 0.1) (Simon GM, Cheng J, Gordon JI 2012 Proc Natl Acad Sci U S A 109:11133-8). The resulting data were used to calculate Pearson and Spearman correlation coefficients (the p-value was calculated using the strongest correlation). **B). Correlation of the transcriptome and proteome data subsets.** Using the significantly affected transcriptome and proteome data, median values were calculated across the samples, and Pearson and Spearman correlation coefficients were determined (mean values resulted in slightly weaker correlations; p was calculated using the strongest correlation). Without q_min_[germ status] filtration, the correlations were weaker but more statistically significant (r_P_ = 0.24, r_S_ = 0.24, p = 7.5E-33).


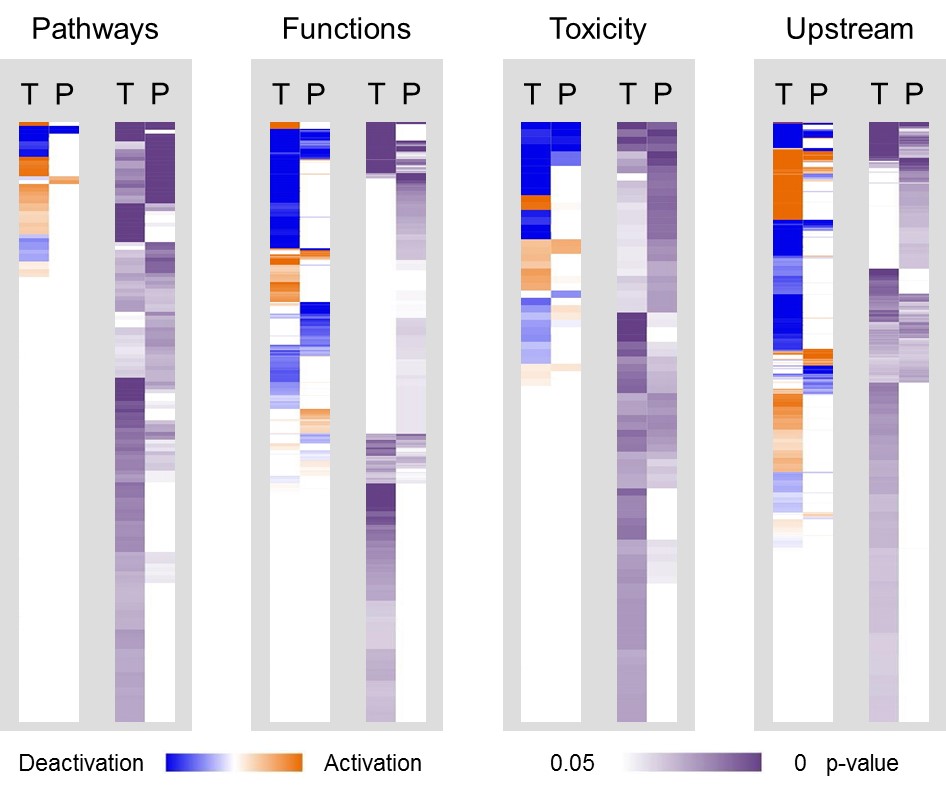


**Figure S4. HCAs of gene annotations affected by germ status**

Ingenuity Pathway Analysis was used to analyze the transcriptome[up + down] and proteome[up + down] gene sets (“T” and “P”, respectively) to discover significantly enriched annotations. Four sets of gene annotations were used: canonical biological pathways, biofunctions, toxicity functions, and upstream regulators. An activation/deactivation value and a p-value was calculated for each annotation. These calculations were independently performed for the transcriptome and proteome, and for each of the four sets of gene annotations. The p-values were not used for multiple hypothesis testing to produce q-values because this capability has not yet been implemented in Ingenuity. Ingenuity was used to produce eight independent HCA heatmaps (each row depicts a gene annotation).

A
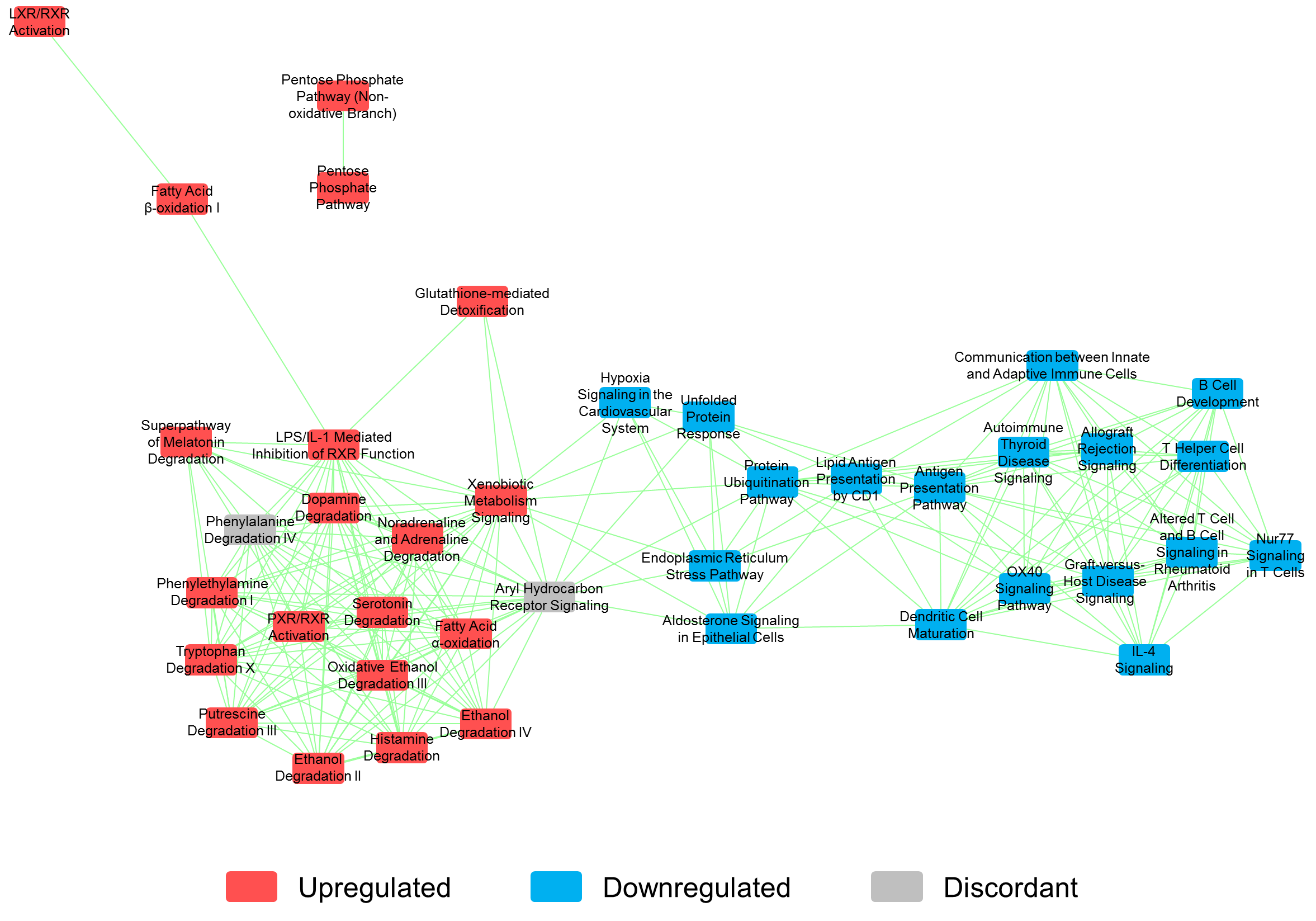


B
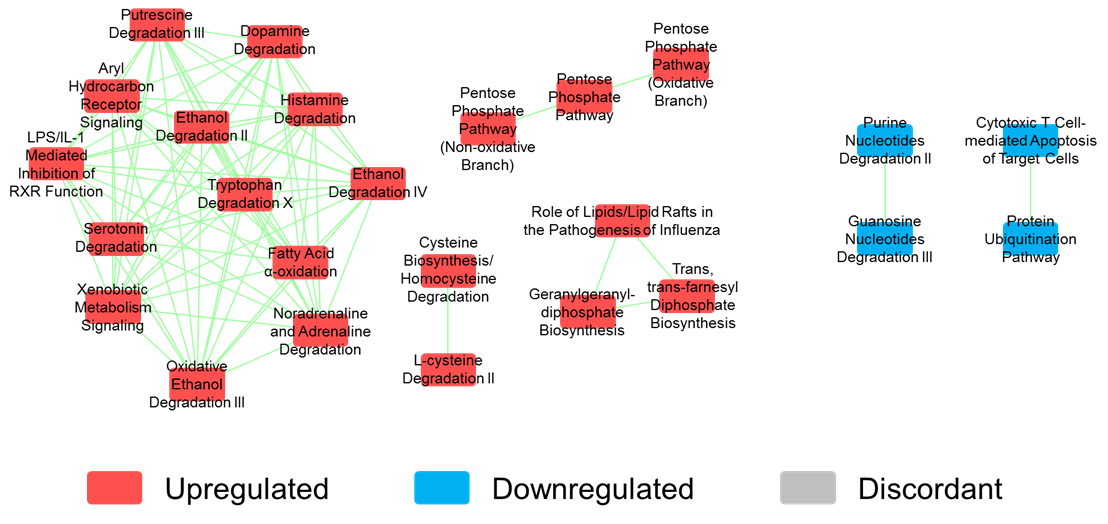


**Figure S5. A). Network analysis of the pathways that were enriched in the proteome gene set.** Each node depicts a significantly enriched biological pathway, and each edge depicts ≥1 overlapping genes that were significantly affected by germ status. This network formed two clusters, one composed of upregulated metabolic pathways (left), and one composed of downregulated immune system pathways (right). **B). Network analysis of the pathways that were enriched in the invariant[P, up] and invariant[P, down] gene sets.** Each node depicts a significantly enriched biological pathway, and each edge depicts ≥1 overlapping genes that were significantly affected by germ status. Two network analyses were performed. The invariant[P, up] analysis resulted in a network of upregulated metabolic pathways (left), and the invariant[P, down] analysis resulted in a small network of downregulated pathways (right).


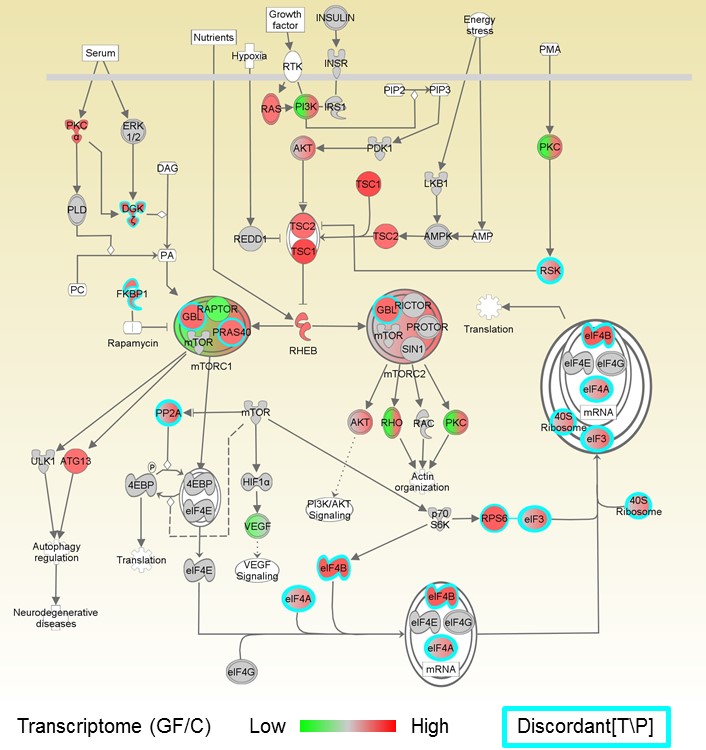


**Figure S6. The mTOR Signaling pathway was intra-set concordant and transcriptome–proteome discordant**

The intra-set concordancy and transcriptome–proteome discordancy were statistically significant (q_e_[transcriptome, up] = 2.9E-06, q_e_[discordant[T\P], up] = 1.5E-02, p_c_[transcriptome] = 4.0E-04). Dual shading of a pathway node depicted data from multiple gene products.


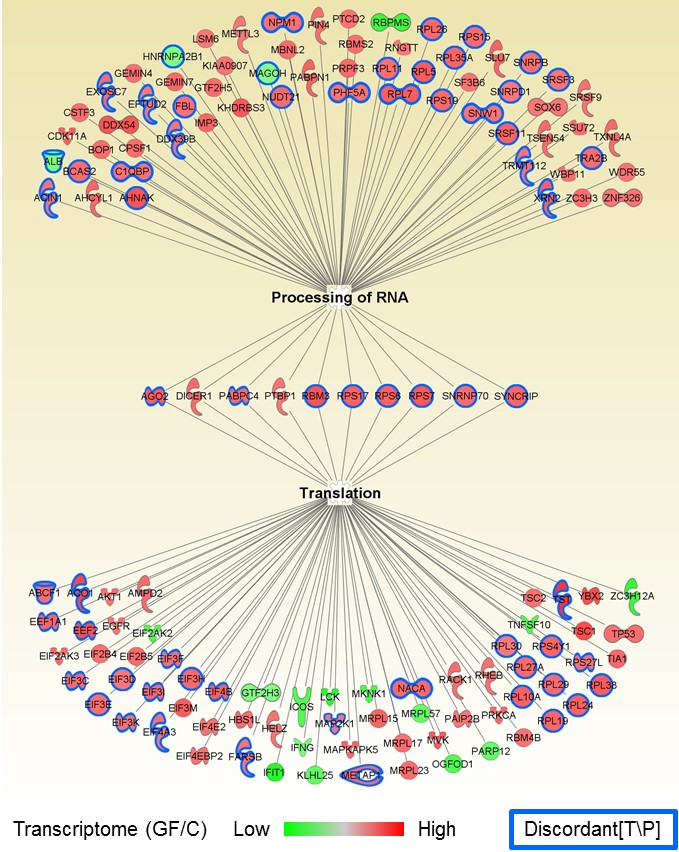


**Figure S7. Intra-set concordancy and transcriptome–proteome discordancy of gene products involved in protein translation.**

Two gene annotations related to protein translation were enriched in the transcriptome data: Processing of RNA (q_e_ = 8.9E-02) and Translation (q_e_ = 8.0E-06). Most of these genes were upregulated (p_c_ = 1.6E-20), and were discordant compared to the proteome data.


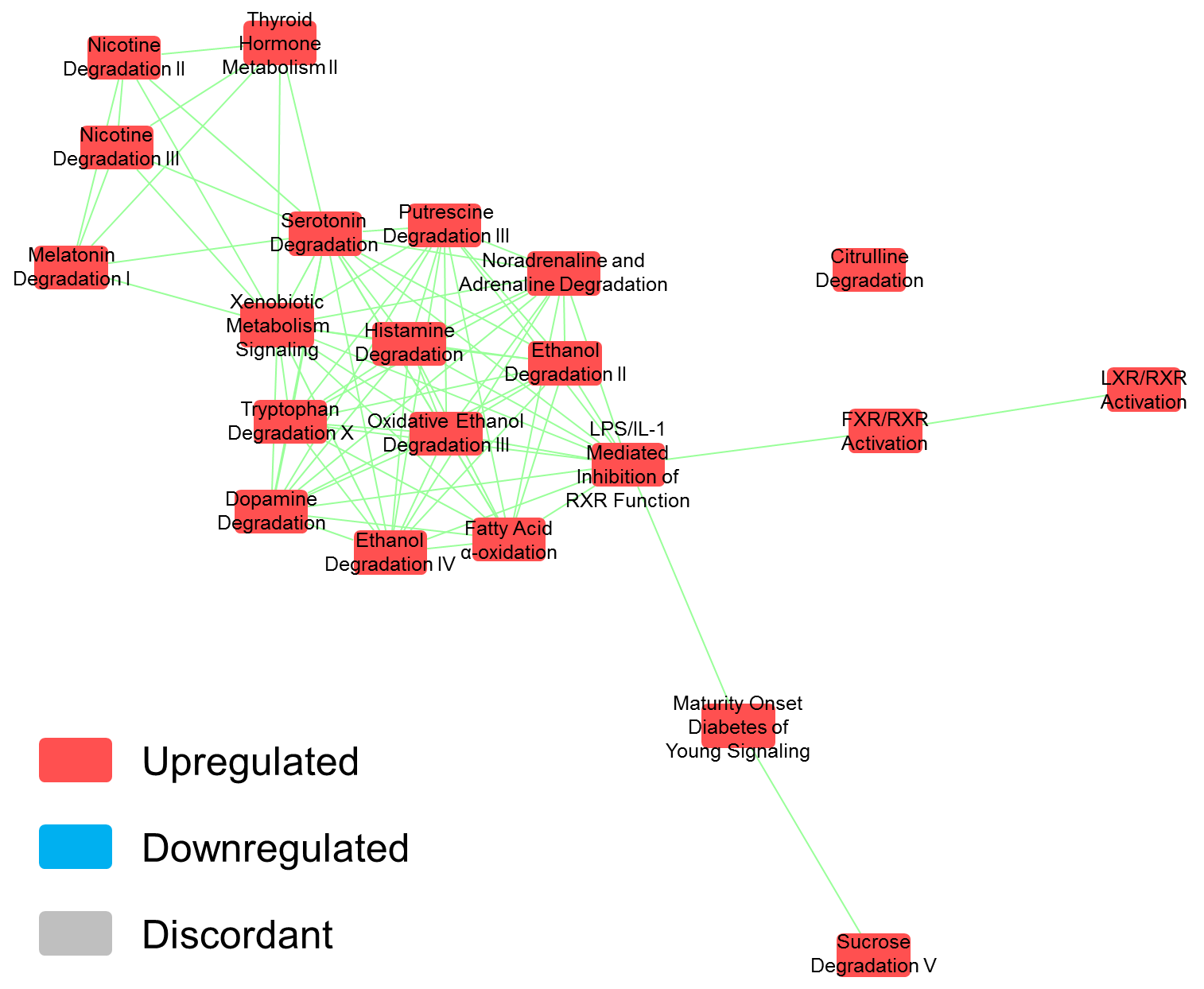


A


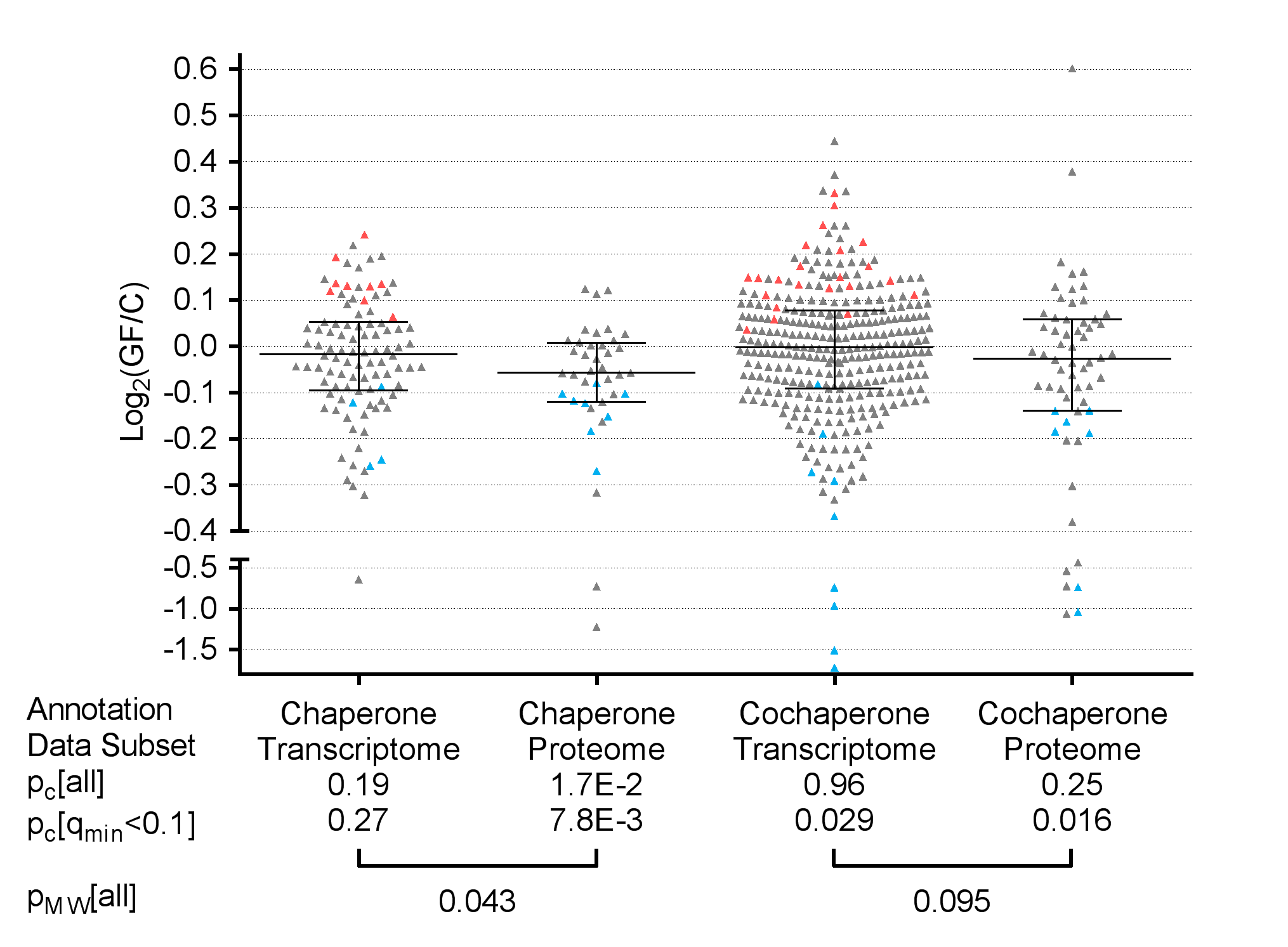


B

**Figure S8. A). Network analysis of the pathways that were enriched in the discordant[P\T, up] gene set.** Each node depicts a significantly enriched biological pathway, and each edge depicts ≥1 overlapping genes that were significantly affected by germ status. This network formed a cluster of upregulated metabolic pathways. **B). Intra-set concordancy and transcriptome–proteome discordancy of (co-)chaperone gene products.** The transcriptome and proteome data related to two annotations were analyzed: chaperones and cochaperones. The statistical tests and plot format are described in the Figure 4C legend.

A


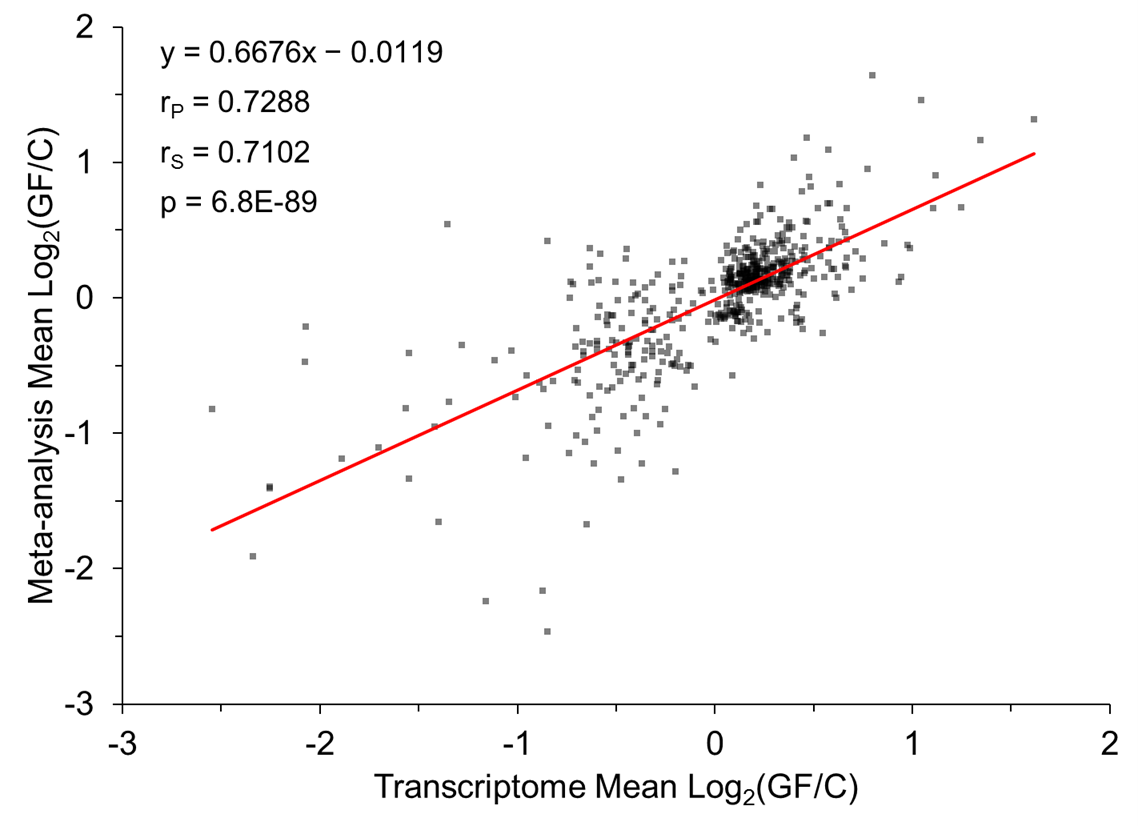


B


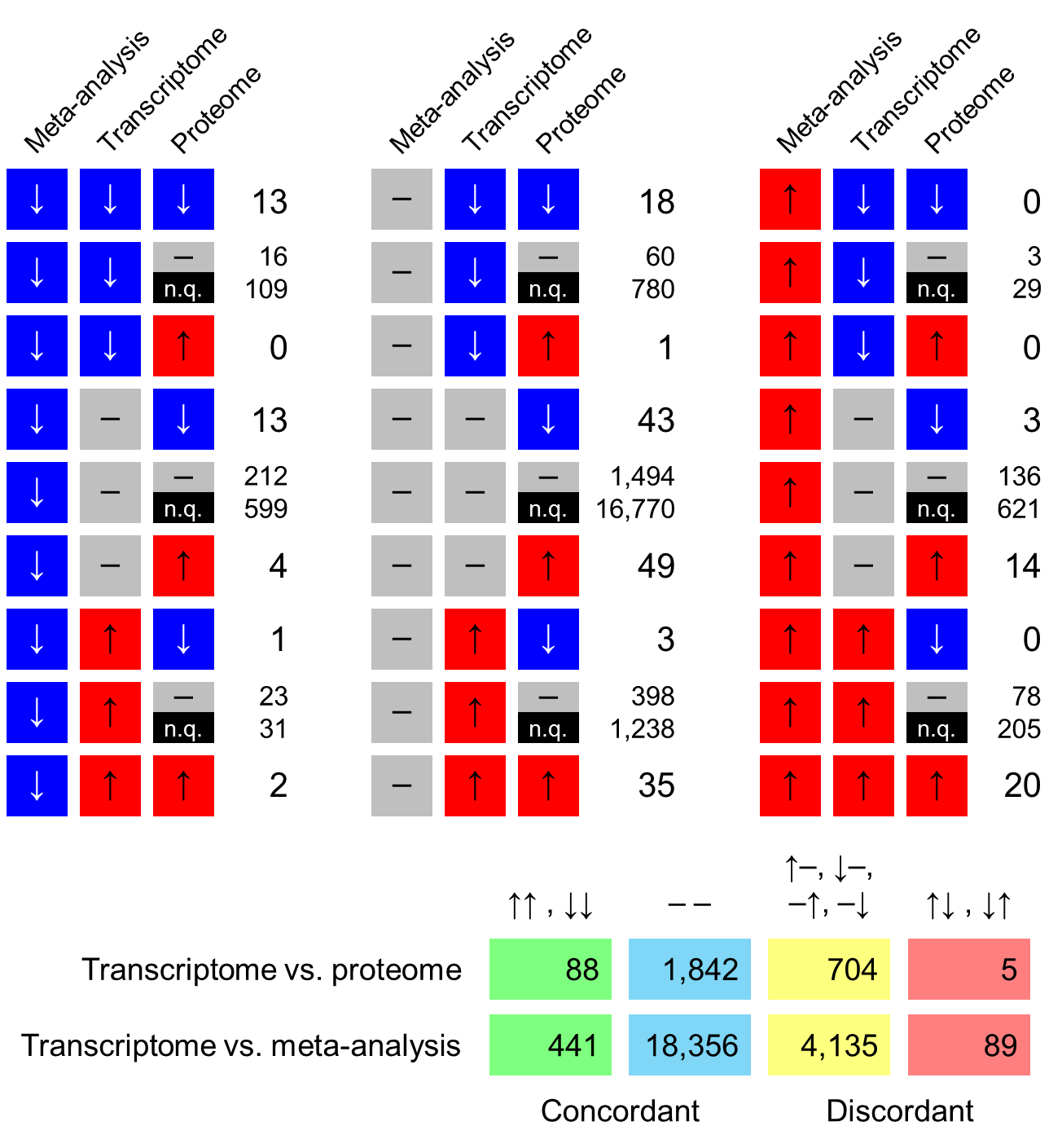


**Figure S9. A). Correlation of the transcriptome and meta-analysis sets of data.** Using the significantly affected transcriptome and meta-analysis data, mean values were calculated across the samples, and Pearson and Spearman correlation coefficients were determined (median values resulted in slightly weaker correlations; p was calculated using the strongest correlation). Without q_min_[germ status] filtration, the correlations were weaker but more statistically significant (r_P_ = 0.50, r_S_ = 0.36, p = 2.8E-252). **B). Tally of the genes across all three sets of data.** For each set of data, genes were tallied based on four categories: significantly upregulated in the GF mice (red), significantly downregulated (blue), unaffected (gray), or unquantified (black). Genes unquantified within the transcriptome and meta-analysis were categorized as unaffected (gray).


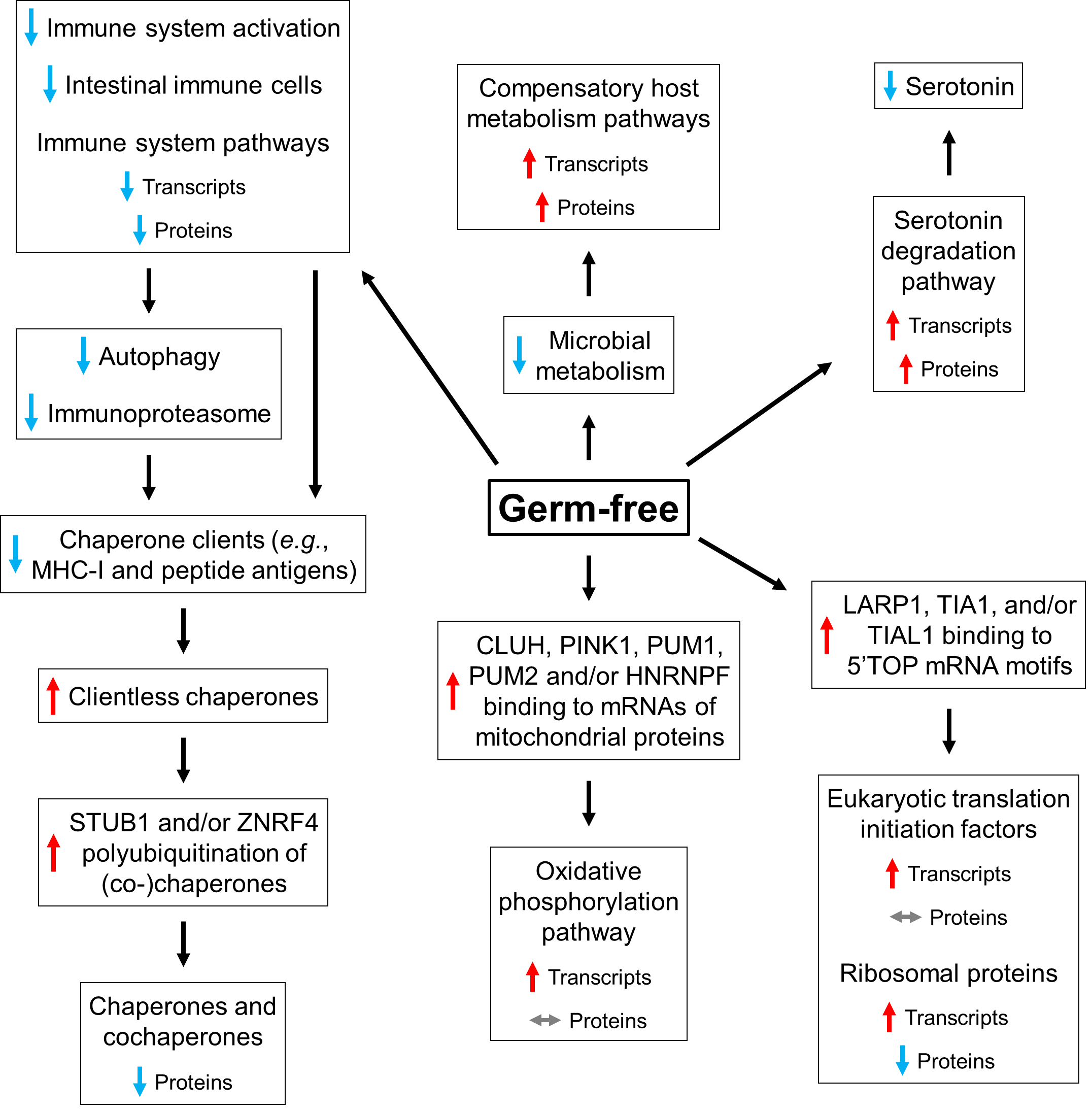


**Figure S10. Model of the observed effects of the microbiota on the host pathways.**

In this hypothetical model, (1) the host metabolic pathway changes are caused by and compensate for the absence of microbial metabolism, (2) increased serotonin degradation explains earlier reports of reduced serotonin in GF mice, (3) the eIF, RP, and OxP changes are caused by posttranscriptional regulation, (4) immune system pathway changes are caused by reduced immune system activation, and (5) reduced immune system activation results in polyubiquitination and degradation of clientless (co-)chaperones. Five serotonin/melatonin metabolic pathways were significantly affected by germ status (Serotonin and Melatonin Biosynthesis, Serotonin Degradation, and Melatonin Degradation I, II, and Superpathway), and this partially agrees with earlier reports (Anderson G, Maes M 2015 Adv Integr Med 2:31-7; Martin AM et al 2017 Endocrinology 158:1049-63). Numerous additional concordances and discordances were observed in the data. For example, at the protein-level eight keratins were significantly affected (KRT4, KRT7, KRT8, KRT13, KRT18, KRT19, KRT20, KRT77), all were upregulated, and all except one (KRT18) were transcriptome–proteome discordant
